# BpOmpW Antigen Stimulates the Necessary Protective T-Cell Responses Against Melioidosis

**DOI:** 10.3389/fimmu.2021.767359

**Published:** 2021-12-13

**Authors:** Julen Tomás-Cortázar, Lorenzo Bossi, Conor Quinn, Catherine J. Reynolds, David K. Butler, Niamh Corcoran, Maitiú Ó Murchú, Eve McMahon, Mahavir Singh, Patpong Rongkard, Juan Anguita, Alfonso Blanco, Susanna J. Dunachie, Daniel Altmann, Rosemary J. Boyton, Johan Arnold, Severine Giltaire, Siobhán McClean

**Affiliations:** ^1^ Conway Institute of Biomolecular and Biomedical Science, University College Dublin, Dublin, Ireland; ^2^ School of Biomolecular and Biomedical Science, University College Dublin, Dublin, Ireland; ^3^ Immunxperts SA, a Nexelis Company, Gosselies, Belgium; ^4^ Department of Infectious Disease, Imperial College London, London, United Kingdom; ^5^ Lung Division, Royal Brompton and Harefield Hospitals, Guy’s and St Thomas’ NHS Foundation Trust, London, United Kingdom; ^6^ LIONEX Diagnostics and Therapeutics GmbH, Brunswick, Germany; ^7^ Mahidol-Oxford Tropical Medicine Research Unit, Bangkok, Thailand; ^8^ Oxford Centre for Global Health Research, University of Oxford, Oxford, United Kingdom; ^9^ Inflammation and Macrophage Plasticity Lab, CIC bioGUNE-BRTA (Basque Research and Technology Alliance), Derio, Spain; ^10^ Ikerbasque, Basque Foundation for Science, Bilbao, Spain

**Keywords:** melioidosis, vaccine, *Burkholderia pseudomallei*, T-cell responses, IFN-γ, type 2 diabetes

## Abstract

Melioidosis is a potentially fatal bacterial disease caused by *Burkholderia pseudomallei* and is estimated to cause 89,000 deaths per year in endemic areas of Southeast Asia and Northern Australia. People with diabetes mellitus are most at risk of melioidosis, with a 12-fold increased susceptibility for severe disease. Interferon gamma (IFN-γ) responses from CD4 and CD8 T cells, but also from natural killer (NK) and natural killer T (NKT) cells, are necessary to eliminate the pathogen. We previously reported that immunization with *B. pseudomallei* OmpW (BpOmpW antigen) protected mice from lethal *B. pseudomallei* challenge for up to 81 days. Elucidating the immune correlates of protection of the protective BpOmpW vaccine is an essential step prior to clinical trials. Thus, we immunized either non-insulin-resistant C57BL/6J mice or an insulin-resistant C57BL/6J mouse model of type 2 diabetes (T2D) with a single dose of BpOmpW. BpOmpW induced strong antibody responses, stimulated effector CD4^+^ and CD8^+^ T cells and CD4^+^ CD25^+^ Foxp3^+^ regulatory T cells, and produced higher IFN-γ responses in CD4^+^, CD8^+^, NK, and NKT cells in non-insulin-resistant mice. The T-cell responses of insulin-resistant mice to BpOmpW were comparable to those of non-insulin-resistant mice. In addition, as a precursor to its evaluation in human studies, humanized HLA-DR and HLA-DQ (human leukocyte antigen DR and DQ isotypes, respectively) transgenic mice elicited IFN-γ recall responses in an enzyme-linked immune absorbent spot (ELISpot)-based study. Moreover, human donor peripheral blood mononuclear cells (PBMCs) exposed to BpOmpW for 7 days showed T-cell proliferation. Finally, plasma from melioidosis survivors with diabetes recognized our BpOmpW vaccine antigen. Overall, the range of approaches used strongly indicated that BpOmpW elicits the necessary immune responses to combat melioidosis and bring this vaccine closer to clinical trials.

## Introduction

Melioidosis is a potentially fatal tropical infection caused by the Gram-negative facultative intracellular bacillus *Burkholderia pseudomallei*. Melioidosis is endemic in Southeast Asia and Northern Australia, but increasingly emerging throughout the tropics. The global incidence is estimated to be 165,000 cases per year with 89,000 deaths and a significant global burden in terms of death and quality of life (1, 2). Infections generally arise from environmental exposure and present as a spectrum of disease ranging from local pathologies such as pneumonia or abscesses to systemic disease and sepsis (3–5). The case fatality rates vary from 35% to 42% in Thailand (6) to 26% recorded in Australia (7). Importantly, individuals with diabetes mellitus (DM) have a 12-fold increased susceptibility to melioidosis and experience more severe disease (5). DM affects over 450 million people worldwide (8), of which 90% are considered to have type 2 diabetes (T2D) (9), and more than 50% of these individuals live in melioidosis endemic regions in Southeast Asia and Northern Australia (1). There is no licensed vaccine available to protect people in endemic regions from melioidosis, including those with T2D.

We previously reported the identification of a protective antigen against melioidosis, identified using a proteomic approach based on the homology between *B. pseudomallei* and *Burkholderia cenocepacia* complex (Bcc) (10, 11). We showed that immunization with the Bcc homologue OmpW in *B. pseudomallei* (BpOmpW) provided protection against two different murine models with distinct genetic backgrounds: BALB/c and C57BL/6J (10). In particular, we showed that 75% of immunized mice survived a lethal challenge for an extended period of 81 days (12), a sustained protection not previously shown for any single subunit vaccine and surpassing that of the live attenuated vaccine 2D2. In comparison, the combination of CPS-CRM197 and Hcp1 (13) protected mice from lethal inhalational challenge for up to 35 days (13).

Understanding the correlates of protection is an essential step in the development of any vaccine (14). Although the necessary protective T-cell responses against melioidosis are poorly understood, it is clear that protection requires competent cellular immune responses mediated by T cells in mice (15), and there is evidence that strongly supports this in humans as well (16). In particular, elevated interferon gamma (IFN-γ) responses associated with CD4^+^ and CD8^+^ T cells are important to combat the disease (15). Moreover, IFN-γ-producing natural killer (NK) and natural killer T (NKT) cells also participate in the response against melioidosis in mice (17) and humans (18, 19). Finally, humoral immunity also contributes to the elimination of *B. pseudomallei* in mice, and protective antibody responses have been described in human observational studies (20, 21).

In order to further evaluate the BpOmpW antigen with a view to progression to human trials, we have undertaken an investigation to elucidate the protective T-cell responses for this vaccine antigen. In particular, we have performed an in-depth analysis of the T-cell responses associated with the BpOmpW antigen in C57BL/6J mice. Moreover, as diabetes is the most important risk factor for severe disease and produces immune function dysregulation (22, 23), we developed an insulin-resistant mouse model of T2D to evaluate the immune responses to the BpOmpW antigen in the context of diabetes as recommended by the Steering Group on Melioidosis Vaccine Development (24). With the aim of progressing to clinical trials, we have examined the IFN-γ responses in HLA-humanized mice, the proliferation of human peripheral blood mononuclear cells (PBMCs) in the presence of the antigen, and the antibody responses against BpOmpW in patients with melioidosis.

## Materials and Methods

### BpOmpW Expression and Purification

The recombinant BpOmpW used in all experiments, except for the enzyme-linked immune absorbent spot (ELISpot) analysis of transgenic mice, was expressed, purified, and provided by Lionex GmBH (Braunschweig, Germany) in 20 mM ammonium bicarbonate. In the case of transgenic mouse studies, the pRSET_BpOmpW construct was transformed in BL21(DE3) cells and cultured in Luria–Bertani (LB) medium with 1 M d-sorbitol and 2.5 mM glycine betaine for 5 days at 22°C. The His-tag fusion protein was then purified by nickel affinity chromatography with endotoxin-free phosphate-buffered saline (PBS), 35 mM imidazole, and 2% Triton X-100 and eluted in endotoxin-free PBS containing 250 mM imidazole and 2% Triton X-100. The antigen was further purified by gel filtration chromatography. The affinity chromatography fraction containing the antigen (as identified by SDS-PAGE) was concentrated and loaded onto a HiLoad 16/600 GL Superdex 75 column (GE Healthcare, Chicago, IL, USA) pre-equilibrated in endotoxin-free PBS using an AKTA chromatography system (GE Healthcare). Fractions with the protein of interest were pooled and the protein was concentrated and stored at −80°C until its use. Protein concentration was determined using the bicinchoninic acid (BCA) protein assay kit (Thermo Fisher Scientific, Waltham, MA, USA) and used for the immunization of transgenic mice and in ELISpot assays.

### Ethics Statement

All work involving animals was approved by the University College Dublin Ethics Committee (AREC-19-13-McClean), and mice were maintained according to the regulations of the Health Products Regulatory Authority (Directive 2010/63/EU and Irish Statutory Instrument 543 of 2012), with authorization number AE18982/P166.

For the human plasma samples from melioidosis patients and controls in Northeast Thailand (*n* = 15, each cohort), the study protocol was approved by the Ethics Committees of the Faculty of Tropical Medicine, Mahidol University (TMEC 12-014); Sunpasitthiprasong Hospital, Ubon Ratchathani (017/2559); and the Oxford Tropical Research Ethics Committee (OXTREC35-15). Blood samples were collected from in-patients with culture-confirmed melioidosis (M), diabetic patients (D), and healthy participants from endemic areas who were household contacts of the melioidosis cases (HH) at Sunpasitthiprasong Hospital, Ubon Ratchathani, Thailand, between 2015 and 2017. All the patients in the M cohort had diabetes. A group of non-endemic individuals (NE; *n* = 3) was also included in the study. Written informed consent was obtained from all patients enrolled in the study.

Cryopreserved primary PBMCs were isolated from whole blood donated by healthy volunteers. Whole blood was collected from healthy donors as described in the ethical protocol IXP-003_V1 (Belgian registration no. B707201627607) or protocol IXP-004_V1 (Netherlands, reg. no. NL57912.075.16). Written informed consent was obtained from all patients enrolled in the study. All blood samples were tested and found negative for hepatitis B virus (HBV), hepatitis C virus (HCV), and human immunodeficiency virus (HIV). PBMCs were separated from the blood by density gradient centrifugation and subsequently cryopreserved in fetal bovine serum (FBS), supplemented with 10% dimethyl sulfoxide, by controlled rate freezing. The PBMCs were kept in cryogenic storage (−180°C) until use.

### Immunization of C57BL/6J Mice for Immunophenotyping

**Figure 1:**
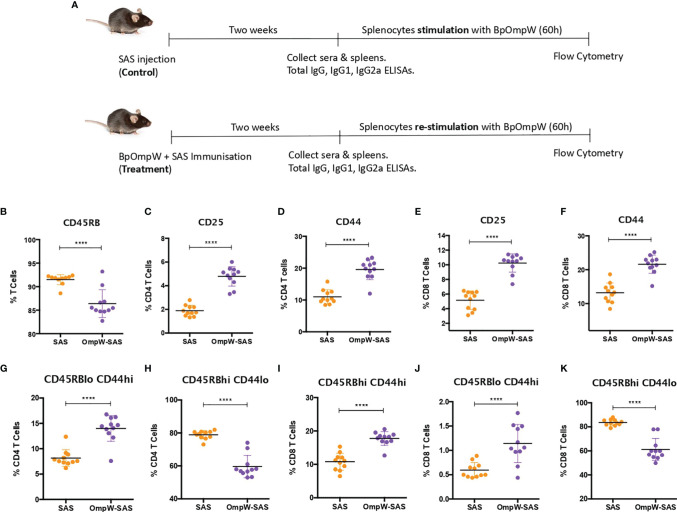
BpOmpW-activated T cells and induced effector CD4^+^ and CD8^+^ T cells. **(A)** Schematic illustration of the experimental timeline of the non-insulin-resistant mouse study. **(B)** Percentages of parent cells expressing CD45RB. **(C–F)** Percentages of CD4 and CD8 T cells expressing CD25 and CD44 activation markers. **(G–K)** Percentages of different populations of CD4 and CD8 T cells defined by different levels of CD45RB and CD44, such as effector CD4 T cells CD45RB^low^CD44^high^ **(G)**, naive CD4 T cells CD45RB^high^CD44^low^ **(H)**, effector CD45RB^high^CD44^high^ CD8 T cells **(I)**, effector CD8 T cells CD45RBlow CD44^high^ **(J)**, and naive CD8 T cells CD45RB^high^CD44^low^ **(K)**. *SAS*, splenocytes from adjuvant-only mice exposed to BpOmpW (*orange circles*, control) (*n* = 11); *OmpW-SAS*, splenocytes from SAS-adjuvanted BpOmpW-immunized mice re-exposed to BpOmpW *in vitro* (*purple circles*, treatment) (*n* = 11); *BpOmpW*, homologue OmpW in *Burkholderia pseudomallei*. *Asterisks* denote statistically significant differences according to a two-tailed *t*-test. The levels of significance are represented as follows: ****p < 0.0001.

Male C57BL/6J mice were used in these studies. Mice were given free access to food and water and subjected to a 12-h light/dark cycle. Groups of C57BL/6J male mice (*n* = 11) were immunized subcutaneously with 50 μg BpOmpW in Sigma Adjuvant System (SAS; Sigma, St. Louis, MO, USA) or SAS adjuvant alone as a negative control (**Figure 1A**). The antigen was mixed with SAS adjuvant at a 1:1 ratio (50:50 μl per mouse). The antigen was diluted in 20 mM ammonium bicarbonate in the same buffer used for purification. Two weeks later, mice were humanely killed by sedation with an isoflurane vaporizer followed by CO_2_ exposure. Blood was withdrawn by cardiac puncture for serological analysis. Sera were extracted from blood and stored at −80°C. Spleens were also harvested and processed for splenocyte re-stimulation assays to assess antigen-specific cell-mediated response to BpOmpW.

### Determination of BpOmpW-Specific IgG Isotypes by ELISA

Microtiter plates were coated with purified BpOmpW at 0.5 μg/ml in sodium bicarbonate buffer (pH 9.4) at 4°C overnight, after which the coating solution was removed and the plates blocked with 1% FBS (7524; Sigma) solution in PBS at room temperature for 1 h. The wells were washed three times with 0.05% Tween 20 in PBS using a plate washer (5165040; Thermo Scientific). All serum samples were serially diluted (fivefold) in PBS containing 1% FBS and 100 μl added to the wells in triplicate at room temperature (RT) for 2 h. The plates were then washed three times with 0.05% Tween 20 in PBS, as described above, before the addition of anti-mouse immunoglobulin G (IgG), IgG1, or IgG2a–horseradish peroxidase (HRP)-conjugated antibodies (ab97023, ab97240, ab97245, respectively; Abcam, Cambridge, UK) at RT for 1 h. Then, the TMB (3,3′,5,5′-tetramethylbenzidine) substrate was added and incubated until the color developed, at which time the reactions were stopped with 2 M sulfuric acid and the plates read at 450 nm. For the detection of BpOmpW-specific IgG in human plasma, the same protocol was applied using anti-human IgG–HRP antibody (ab6858; Abcam) in place of anti-mouse IgG antibodies. All the HRP-conjugated antibodies were diluted 1:5000 in PBS containing 1% FBS. The color intensity at OD_450_ correlated with the amount of specific antibodies in the sera.

### Splenocyte Re-Stimulation With BpOmpW Antigen

The spleen from each mouse was mechanically disrupted and filtered to obtain a single-cell suspension of splenocytes. Then, an ammonium–chloride–potassium (ACK) lysis buffer was used to remove red blood cells. The splenocytes from BpOmpW-immunized or control mice were then counted automatically using a Countless automated Cell Counter (Invitrogen, Carlsbad, CA, USA). One million cells in triplicate (per mouse) were plated per well and stimulated for 60 h with 50 μg/ml BpOmpW in a 96-well-plate using 10% FBS RPMI medium and 1% P/S (Penicillin-Streptomycin). Between 5 and 6 h before harvesting the cells, 5 μg/ml brefeldin A was added to block cell trafficking in order to increase the accumulation of intracellular cytokines. Cells were collected by centrifugation and stained for flow cytometry. Studies in non-insulin resistant and insulin resistant mice were performed independently at different times.

### Flow Cytometry

The stimulated splenocytes were incubated with Fc Block (anti-CD16/CD32; BD Biosciences, Franklin Lakes, NJ, USA) for 5 min on ice and labeled with Viakrome 808 (Beckman Coulter, Brea, CA, USA) and the fluorochrome-labeled antibodies against CD4, CD49b, CD45RB, CD8a, CD25, CD44, and CD3 surface markers (BD Biosciences) for 30 min on ice. Then, intracellular interleukin 2 (IL-2), IL-4, IFN-γ, IL-17, IL-9, tumor necrosis factor (TNF), and Foxp3 (BD Biosciences) were also analyzed with a BD Cytofix/CytoPerm™/Fixation/Permeabilization Solution Kit (BD Biosciences) according to the manufacturer’s instructions. The antibodies were all titrated for optimal performance, and Beckman Coulter VersaComp beads were used for compensation. A Beckman Coulter CytoFLEX LX (NUV full configuration) was used for the analysis of the samples with the gains for each one of the markers optimized by performing a gaintration. Quality control of the instrument was performed using Beckman Coulter Daily QC beads and IR Daily QC as per the manufacturers’ specifications. Fluorescence minus one (FMO) controls were used for every single marker in order to generate the gates, and several FMO controls were used daily for verification purposes. The gating strategy and the corresponding FMO controls for each gate are shown in **Figures S3–S9**. Data analysis and reanalysis were performed with Beckman Coulter CytExpert v.2.4. The supplementary Excel file shows the full MIFlowCyt (the minimum information about a flow cytometry experiment). Data were extracted as the percentage of parental cells.

### Polygenic Insulin-Resistant Mouse Model

Seventy C57BL/6J male mice were fed a D12492i rodent diet comprising 60 kcal% from fat (Research Diets, Inc., New Brunswick, NJ, USA) or regular chow starting at 6–8 weeks of age until they were humanely killed. Insulin resistance was determined at 8 and 12 weeks by fasting the mice for 6 h, at which time 0.5 U/kg of insulin (Actrapid; Novo Nordisk, Bagsværd, Denmark) was administered subcutaneously. In order to alleviate any pain, EMLA cream was applied to the whole tail 10 min before blood was sampled from the tail vein using a 27-gauge needle, a drop of blood extracted at 15, 30, 45 and 60 min, and the glucose levels measured using the AlphaTRAK^®^ 2 blood glucose monitor. To characterize the model, we collected the spleen, liver, pancreas, and kidney from the mice and a hematoxylin–eosin (H&E) stain was applied to the cut tissue sections. The time required for individual mice to develop insulin resistance varied, and only mice that showed insulin resistance were considered eligible to be randomly selected for immunization studies.

### ELISpot Analysis of IFN-γ Recall Response to Antigen or Peptides

The HLA-DR and HLA-DQ alleles are associated with melioidosis. This study used HLA class II transgenic mouse lines for the alleles HLA-DR1 (DRB1*0101), HLA-DR4 (DRB1*0401), and HLA-DQ8 (DQB1*0302), which were, in each case, maintained in the context of a homozygous knockout for murine H2-Ab. Mice were maintained in individually ventilated cages and were used in experiments as age- and sex-matched young adults. For CD4 T-cell epitope mapping studies, the mice were primed in one hind footpad with 25 μg antigen emulsified in Hunters Titermax Gold adjuvant (Sigma-Aldrich, St. Louis, MO, USA). On day 10, the draining popliteal lymph node was removed and disaggregated into a single-cell suspension for ELISpot assays. The frequency of cells producing IFN-γ in response to the antigen was quantified with ELISpot (Diaclone; 2B Scientific, Oxon, UK) performed in HL-1 serum-free medium (BioWhittaker; Lonza, Slough, UK) supplemented with l-glutamine and penicillin–streptomycin (Life Technologies, Paisley, UK). Cells plus antigen were added to the wells and plates and were incubated for 72 h at 37°C with 5% CO_2_. Unless otherwise indicated, peptide was added to the wells at a final concentration of 25 μg/ml. Spots were counted on an automated ELISpot reader (Autoimmun Diagnostika, Strasbourg, France). Response frequencies were expressed as ΔSFC/10^6^ cells, with the presence of an epitope being confirmed when the majority of the mice in the immunized group responded with a magnitude greater than the mean number of spot-forming cells (SFCs) in medium-only control + 2 SD. The mean + 2 SD background SFCs for murine ELISpot data are indicated in each case by a dotted line in the figures. The ELISpot background range (per 10^6^ cells) was 0–30 SFCs.

### PBMC Proliferation Assay

PBMCs from 23 healthy human donors were thawed from ImmunXperts biobank and incubated for 7 days at 37°C with BpOmpW (50 µg/ml), SAS (1 µg/ml), and their combination in six replicates. T-cell proliferation was assessed by measuring the incorporation of 5-ethynyl-2′-deoxyuridine (EdU). EdU is a thymidine analogue incorporated into the DNA of dividing cells during the S-phase. On day 6 of culture, the cells were pulsed with EdU at 1 µM for 16 h. The next day, cell culture supernatants from human PBMC stimulation assays were collected and stored at −80°C for cytokine analysis. The remaining cells were fluorescently stained for T-cell surface markers (CD4 and CD8), fixed, permeabilized, and the incorporated EdU was stained with a fluorescent azide to finally perform the analysis using flow cytometry. The gating strategy for this experiment can be found in **Figure S11**.

### Human IFN-γ ELISA

The supernatant was stored at −80°C and then analyzed for the quantification of IFN-γ with LEGEND MAX™ Human IFN-γ ELISA Kit (BioLegend, San Diego, CA, USA) following the manufacturer’s instructions.

### Statistical Analysis

The sample size in all cases was determined using G*Power software. The results are presented as mean ± SE, unless otherwise stated. Differences in the means between groups were tested using a *t*-test or ANOVA using GraphPad Prism, version 7. A *p*-value <0.05 was considered statistically significant.

## Results

### BpOmpW Immunization Activated T Cells and Induced Effector CD4^+^ and CD8^+^ T Cells

T-cell-inducing vaccines are required to induce CD4^+^ and/or CD8^+^ T cells of sufficient effector function that directly participate in pathogen removal *via* cell-mediated effector mechanisms (25). Therefore, given that T-cell effector functions, especially IFN**-**γ responses, correlate with survival in acute melioidosis patients (16), we examined the T-cell responses associated with BpOmpW immunization. Groups of 11 mice were immunized once with recombinant BpOmpW (**Figure 1**). Analysis of the mouse sera following BpOmpW immunization showed strong seroconversion (total IgG, IgG1, and IgG2a) at 2 weeks despite the mice receiving only one immunization (**Figures S1A–C**). Antigen-specific T-cell responses were then determined in splenocytes that had been re-stimulated (treatment group) to antigen *in vitro* by measuring the cytokine responses and a range of T-cell markers using flow cytometry. These were compared to antigen-exposed splenocytes from the adjuvant-only group (SAS control) (**Figure 1A**). Activation of BpOmpW-re-stimulated splenocytes was demonstrated by a significant decrease in CD45RB expression (*p* < 0.0001; **Figure 1B**), while the levels of CD25 and CD44 were significantly increased in response to BpOmpW re-exposure in both CD4^+^ and CD8^+^ T cells when compared to splenocytes from SAS control mice (*p* < 0.0001; **Figures 1C–F**). To further analyze the activation of T cells in immunized mice, we evaluated the relative expression of CD45RB and CD44 in both CD4^+^ and CD8^+^ T cells. A greater number of effector compared to naive CD4^+^ and CD8^+^ T cells were observed in splenocytes from BpOmpW-immunized mice compared to controls. Effector CD4^+^ CD45RB^lo^CD44^hi^ T cells were overrepresented in antigen-re-exposed splenocytes (*p* < 0.0001; **Figure 1G**). Consistent with this, the naive CD4^+^ CD45RB^hi^CD44^lo^ subset was significantly reduced in the BpOmpW-immunized group relative to SAS control splenocytes (*p* < 0.0001; **Figure 1H**). Likewise, both effector CD8^+^ T cells CD45RB^hi^CD44^hi^ (**Figure 1I**) and CD45RB^lo^CD44^hi^ (**Figure 1J**) were increased following BpOmpW immunization compared to SAS controls, whereas the number of naive CD45RB^hi^CD44^lo^ CD8^+^ T cells were reduced (**Figure 1K**). These data show that immunization with BpOmpW results in the generation of strong antigen-specific CD4^+^ and CD8^+^ T-and B-cell responses.

**Figure 2:**
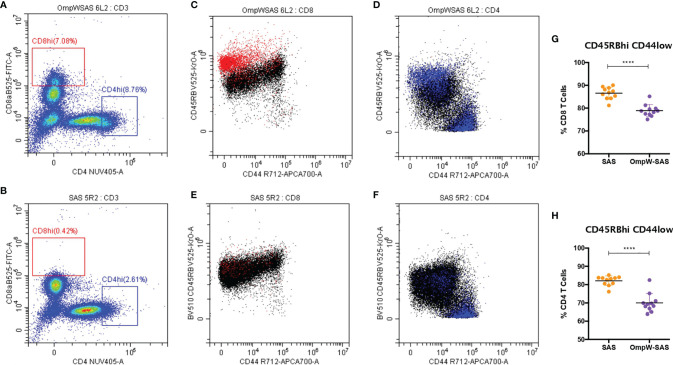
BpOmpW-stimulated CD8^hi^ and CD4^hi^ populations that are CD45RB^hi^CD44^lo^. **(A, B)** Splenocytes of CD8^hi^ (in *red*) and CD4^hi^ (in *blue*) populations from both SAS-adjuvanted BpOmpW-immunized mice re-exposed to BpOmpW (*n* = 11) **(A)** and from adjuvant-only mice exposed to BpOmpW (*n* = 11) **(B)**. **(C, D)** CD4 **(C)** and CD8 **(D)** T-cell co-expressions of CD45RB and CD44 in BpOmpW-re-exposed splenocytes from SAS-adjuvanted BpOmpW-immunized mice. **(E, F)** CD4 **(E)** and CD8 **(F)** T-cell co-expressions of CD45RB and CD44 in BpOmpW-exposed splenocytes from SAS-adjuvanted saline-immunized mice. **(G, H)** Naive CD4 and CD8 T cells (CD45RB^hi^CD44^lo^) including CD45RB^veryhi^ populations as naive T cells. *BpOmpW*, homologue OmpW in *Burkholderia pseudomallei*; *SAS*, Sigma Adjuvant System. The levels of significance are represented as follows: ****p < 0.0001.

We also investigated the CD4^+^ and CD8^+^ T-cell populations in more detail, and we observed that BpOmpW immunization induced the appearance of CD8^hi^ and CD4^hi^ populations (**Figure 2A**) that were absent in the control group splenocytes (**Figure 2B**). Further analysis of these populations demonstrated that they were predominantly CD8^hi^CD45RB^veryhi^CD44^lo^ (**Figure 2C**, in red) and CD4^hi^CD45RB^veryhi^CD44^lo^ subpopulations (**Figure 2D**, in blue), in contrast to SAS control splenocytes that did not show these subpopulations (**Figures 2E, F**). Furthermore, splenocytes from SAS control mice showed a CD4^hi^ population (**Figure 2B**), to a lesser extent than the BpOmpW group, although the population was predominantly CD45RB^lo^CD44^hi^ (**Figure 2F**). It is important to highlight that, including these CD45RB^veryhi^CD44^lo^ populations as if they were CD45RB^hi^CD44^lo^ naive CD4^+^ or CD8^+^ T cells, these subsets were still lower in the BpOmpW-immunized group compared to the SAS control group in both CD8^+^ (**Figure 2G**) and CD4^+^ (**Figure 2H**) T cells.

### BpOmpW Immunization Stimulated IFN-γ Responses in CD4^+^, CD8^+^, NK, and NKT Cells and Increased Regulatory T Cells

**Figure 3:**
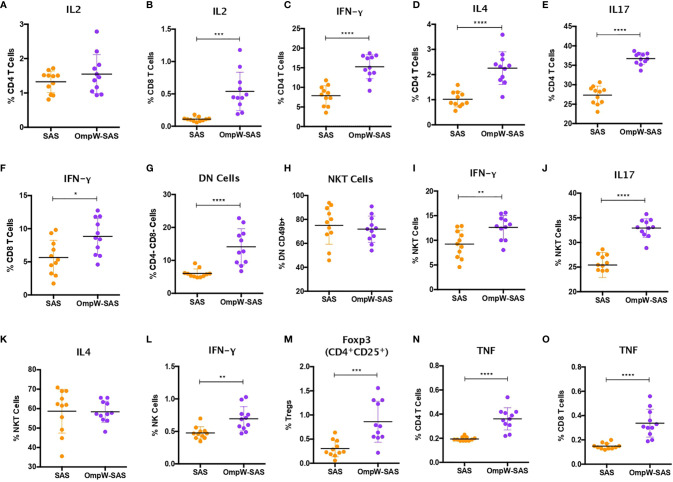
BpOmpW immunization stimulated IFN-γ responses in CD4^+^, CD8^+^, natural killer (NK), and natural killer T (NKT) cells and upregulated regulatory T cells (Tregs). **(A, B)** Percentages of CD4 **(A)** and CD8 **(B)** T cells expressing IL-2 cytokine. **(C–E)** Percentages of CD4 T cells expressing IFN-γ **(C)**, IL-4 **(D)**, and IL-17 **(E)**. **(F)** Percentages of CD8 T cells expressing IFN-γ. **(G)** Percentages of double-negative (DN) cells (CD4^−^CD8^−^). **(H)** Percentages of NKT cells. **(I–K)** Percentages of NKT cells expressing IFN-γ **(I)**, IL-17 **(J)**, and IL-4 **(K)**. **(L)** Percentages of NK cells expressing IFN-γ. **(M)** Percentages of Tregs. **(N, O)** Percentages of CD4 **(N)** and CD8 **(O)** T cells expressing TNF. *SAS*, splenocytes from adjuvant-only mice exposed to BpOmpW (*orange circles*, control) (*n* = 11); *OmpW-SAS*, splenocytes from SAS-adjuvanted BpOmpW-immunized mice re-exposed to BpOmpW *in vitro* (*purple circles*, treatment) (*n* = 11). *Asterisks* denote statistically significant differences according to a two-tailed *t*-test. The levels of significance are represented as follows: *p < 0.05; **p < 0.01; ***p < 0.001; ****p < 0.0001.

IFN-γ responses dominate the immune response induced by melioidosis infection in patients (26). Consequently, in order to elucidate the T-cell responses associated with BpOmpW immunization, we evaluated a range of cytokine levels by flow cytometry following re-stimulation *in vitro*. Although the autocrine growth factor IL-2 did not change in CD4^+^ T cells, it was upregulated in CD8^+^ T cells from the BpOmpW-immunized group relative to the SAS control group (**Figures 3A, B**). The antigen elicited high levels of IFN-γ, IL-4, and IL-17, indicating that CD4^+^ T cells differentiated to Th1, Th2, and Th17 cells, indicative of a mixed T helper (Th) response being elicited by BpOmpW (*p* < 0.0001, *p* < 0.0001, and *p* < 0.0001, respectively; **Figures 3C–E**). Additionally, IFN-γ-producing CD8^+^ T cells were also more abundant following BpOmpW immunization (*p* = 0.011; **Figure 3F**).

NK and NKT cells have been associated with patient responses to melioidosis *(* 18, 19). Consequently, we evaluated the responses of NK and NKT cells in BpOmpW-re-exposed splenocytes. A population of T cells that were negative for both CD4 and CD8, i.e., double-negative (DN) cells, were elevated in the BpOmpW-immunized group relative to the SAS control group (*p* = 0.0001; **Figure 3G**). The expression of CD49b^+^ indicated that NKT cells constituted virtually all these DN cells (**Figure 3H**). Moreover, the IFN-γ and IL-17 responses produced by NKT cells were increased in BpOmpW-immunized mice in comparison with the SAS-adjuvanted control mice, in contrast to IL-4 that remained comparable to SAS control splenocytes (*p* = 0.0062, *p* < 0.0001, and *p* = 0.942, respectively; **Figures 3I–K**). Finally, IFN-γ-producing CD49b^+^ NK cells were also significantly higher in splenocytes from BpOmpW-immunized mice following re-exposure to antigen, relative to the adjuvant-only control group (*p* = 0.0025; **Figure 3L**).

We also examined regulatory T cells as they can suppress pro-inflammatory damage produced by bacterial infections. CD4^+^CD25^+^Foxp3^+^ regulatory T cells were augmented in response to BpOmpW re-exposure in immunized mice relative to the spleen cells from adjuvant-only control mice (*p* = 0.0007; **Figure 3M**). Finally, many studies have observed elevated expression of TNF during melioidosis infection in humans and animal models (27, 28). In this study, TNF was upregulated in both CD4^+^ and CD8^+^ T cells from BpOmpW-immunized mice (*p* < 0.0001 and *p* < 0.0001, respectively; **Figures 3N, O**).

### The Immune Response of Insulin-Resistant Mice to BpOmpW Was Predominantly Comparable to That of Non-Insulin-Resistant Mice

Due to the exquisitely enhanced susceptibility of people with diabetes to infection, and T2D being on the rise in tropical and subtropical regions, we needed to understand immune response in the context of diabetes. We developed a polygenic insulin-resistant mouse model by feeding C57BL/6J male mice with a high-fat diet (HFD) for up to 16 weeks as a model of T2D. Mice on HFD continuously gained more weight than did their littermates on a normal diet from the first 2 weeks of diet intervention (25.49 ± 2.12 *vs*. 29.38 ± 0.82, *p* = 0.00001) to 12 weeks (**Figure S2B**). Moreover, starting at week 8, HFD-fed mice began to develop hyperglycemia (HG = 13.17 ± 2.16 *vs*. 15.66 ± 2.77, *p* = 0.02) and insulin resistance (IR) (*t*_45_ = 46.52 ± 16.26 *vs*. 59.89 ± 23.20, *p* = 0.13), the latter being more apparent at 12 weeks of treatment with the diet (HG = 11.44 ± 1.89 *vs*. 13.82 ± 3.11, *p* = 0.044; *t*_45_ = 44.19 ± 8.20 *vs*. 98.32 ± 17.92, *p* < 0.00001) (**Figures S2C–E**). In addition, large lipid droplets were observed in the livers of insulin-resistant mice relative to control mice in the liver micrographs (**Figure S2F**).

**Figure 4:**
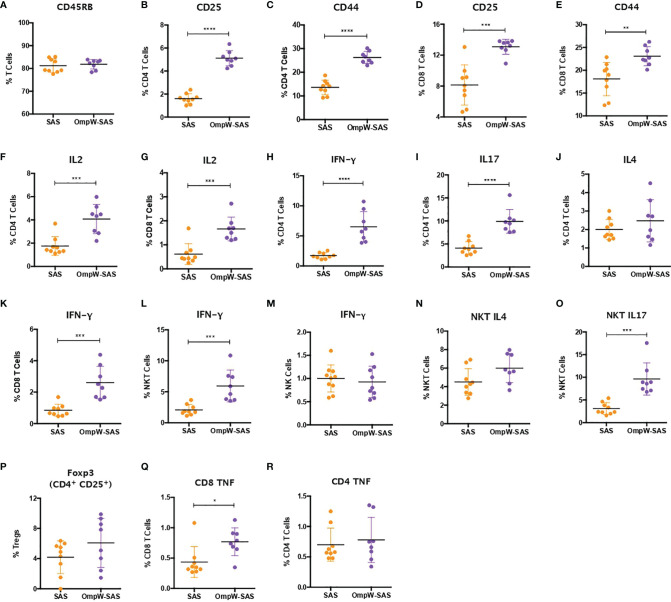
Insulin-resistant mice mimicked with minimal changes the immune response to BpOmpW (homologue OmpW in *Burkholderia pseudomallei*) produced by non-insulin-resistant mice. **(A)** Percentages of the CD45RB marker in total T cells. **(B–E)** Percentages of CD4 and CD8 T cells expressing CD25 and CD44 activation markers. **(F, G)** Percentages of CD4 **(F)** and CD8 **(G)** T cells expressing IL-2 cytokine. **(H–J)** Percentages of CD4 T cells expressing IFN-γ **(H)**, IL-17 **(I)**, and IL-4 **(J)**. **(K–M)** Percentages of CD8 **(K)**, NKT **(L)**, and NK **(M)** cells expressing IFN-γ. **(N, O)** Percentages of NKT cells expressing IL4 **(N)** and IL17 **(O)**. **(P)** Percentages of regulatory T cells (Tregs). **(Q, R)** Percentages of CD4 **(Q)** and CD8 **(R)** T cells expressing the tumor necrosis factor (TNF) cytokine. *SAS*, splenocytes from adjuvant-only mice exposed to BpOmpW (*orange circles*, control) (*n* = 11); *OmpW-SAS*, splenocytes from SAS-adjuvanted BpOmpW-immunized mice re-exposed to BpOmpW *in vitro* (*purple circles*, treatment) (*n* = 11). *Asterisks* denote statistically significant differences according to a two-tailed *t*-test. The levels of significance are represented as follows: *p < 0.05; **p < 0.01; ***p < 0.001; ****p < 0.0001.

To determine the impact of insulin resistance on the response to BpOmpW immunization, groups of 10 insulin-resistant mice (**Figure S3A**) were immunized with one subcutaneous injection of adjuvant alone or with SAS-adjuvanted BpOmpW as before. After 2 weeks, the splenocytes of both groups were exposed to BpOmpW and immunophenotyped. Unexpectedly, CD45RB was not decreased in the insulin-resistant model (*p* = 0.6198; **Figure 4A**) despite the T-cell activation markers, CD25 and CD44, being consistently elevated in both CD4^+^ and CD8^+^ T cells in the BpOmpW-immunized group with respect to the SAS control group (*p* < 0.0001, *p* < 0.0001, *p* = 0.0001, and *p* = 0.0039, respectively; **Figures 4B–E**), which was comparable to that in non-insulin-resistant mice. Insulin-resistant BpOmpW-immunized mice showed elevated levels of IL-2 in both CD4^+^ and CD8^+^ T cells following exposure to the vaccine antigen relative to the splenocytes from adjuvant control mice (*p* = 0.0004 and *p* = 0.0003, respectively; **Figures 4F, G**), in contrast to non-insulin-resistant mice which only showed upregulation of IL-2 in CD8^+^ T cells (**Figure 3B**). Splenocyte cytokine responses from BpOmpW-immunized insulin-resistant mice showed upregulated Th1 and Th17 responses as determined by the levels of IFN-γ and IL-17 in CD4+ T cells, respectively (*p* < 0.0001 and *p* < 0.0001, respectively; **Figures 4H, I**). In contrast to non-insulin-resistant mice, the Th2 response-associated IL-4 levels in CD4^+^ T cells were unaltered with respect to the control group (*p* = 0.2919; **Figure 4J**). Remarkably, after antigen re-stimulation, IFN-γ-producing cytotoxic CD8 and NKT cells were also increased in BpOmpW-immunized insulin-resistant mice relative to the SAS control group (*p* = 0.0005 and *p* = 0.0013, respectively; **Figures 4K, L**), with the exception of IFN-γ-producing NK cells which remained unchanged (*p* = 0.6148; **Figure 4M**). Moreover, no significant changes were observed in IL-4- or IL-17-expressing NKT cells (**Figures 4N, O**). In contrast to non-insulin-resistant mice, the levels of regulatory T cells remained unmodified (*p* = 0.1729) in the splenocytes from BpOmpW-immunized mice relative to SAS control splenocytes (**Figure 4P**). The TNF in CD4 T cells was unchanged relative to the control group in the insulin resistance study (**Figure 4Q**), whereas the TNF from CD8^+^ T cells was more abundant in the BpOmpW group compared to control cells (*p* = 0.012; **Figure 4R**). We also analyzed the antibody responses and the naive and effector T cells in the insulin-resistant model. We observed strong seroconversion (**Figures S3B–D**) and elevated effector T cells in the presence of BpOmpW (**Figures S3E–I**), as in non-insulin-resistant mice.

### BpOmpW Elicited the IFN-γ Recall Responses in Humanized HLA-DR and HLA-DQ Transgenic Mice

**Figure 5:**
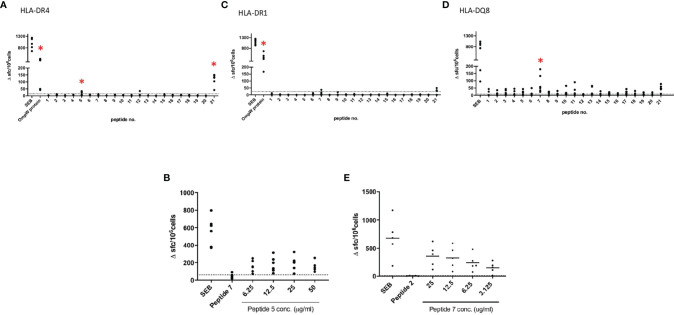
BpOmpW (homologue OmpW in *Burkholderia pseudomallei*) induced the production of IFN-γ in HLA-DR and HLA-DQ (human leukocyte antigen DR and DQ isotypes, respectively) transgenic mice. Immunization of HLA-DR and HLA-DQ transgenic mice highlights HLA class II determining immunodominant epitopes of BpOMpW. **(A–C)** Mice transgenic for HLA-DR4 (*n* = 6), **(A)**, HLA-DR1 (*n* = 6) **(B)**, and HLA-DQ8 (DQB1*0302) (*n* = 6) **(C)** were primed with 25 μg rBpOmpW and the draining lymph node cells assayed with IFN-γ ELISpot in response to the indicated peptide on day 10. Data are plotted as spot-forming cells (SFCs) per 10^6^ cells for individual mice. **(D, E)** Responses to peptide were defined as positive if SFCs are greater than the mean + 2 SD of the response in the absence of any antigen (shown as *horizontal dotted line*): *n* = 6 **(D)** and *n* = 5 **(E)** transgenic mice. The levels of significance are represented as follows: *p < 0.05.

This and our previous work clearly demonstrated that BpOmpW antigen is immunogenic and elicits protective T-cell responses in mice; thus, as a precursor to its evaluation in human studies, we wanted to examine the responses in humanized HLA transgenic mice expressing different HLA alleles. The IFN-γ responses to the whole antigen were examined for epitopes in the context of HLA-DR1, HLA-DR4, and HLA-DQ8 alleles. In addition, a synthetic panel of overlapping peptides was generated covering the full coding sequence (**Table 1**), and these were also evaluated in humanized transgenic mice. The lymph nodes of HLA class II transgenic mice (HLA-DR1, HLA-DR4, and HLA-DQ8) that had been immunized with recombinant BpOmpW antigen showed strong recall responses to the whole BpOmpW antigen and to immunodominant T-cell epitopes, P5, P7, and P21, as determined by the IFN-γ ELISpot assay (**Figure 5**). When probed further by the priming of additional HLA-DR4 transgenic mice with P5 or P21, ELISpot analysis of drain lymph node cells confirmed that peptide 5 was a T-cell epitope. Subsequent priming of additional HLA-DQ8 mice with P7 confirmed that, in HLA-DQ8 transgenics, P7 was a T-cell epitope (**Figure 5**).

**Table 1:** BpOmpW BPSL1552 peptide panel (accession no. CAH35553.1).

Peptide	Peptide name	AA sequence
1	BPSL1552 (1–20)	MRRQTIRTCTTAIACAAGLA
2	BPSL1552 (11–30)	TAIACAAGLAMIPSLSHAAS
3	BPSL1552 (21–40)	MIPSLSHAASPGEGINQGDI
4	BPSL1552 (31–50)	PGEGINQGDIIARVRGISIM
5	BPSL1552 (41–60)	IARVRGISIMPDERTSNTLS
6	BPSL1552 (51–70)	PDERTSNTLSALNVGVNNAI
7	BPSL1552 (61–80)	ALNVGVNNAIVPELDFTYMI
9	BPSL1552 (71–90)	VPELDFTYMIRDYLGVELIL
10	BPSL1552 (81–100)	RDYLGVELILGTSRHQITSS
11	BPSL1552 (91–110)	GTSRHQITSSLGDLGGVGVL
12	BPSL1552 (101–120)	LGDLGGVGVLPPTLLLQYHF
13	BPSL1552 (111–130)	PPTLLLQYHFNHAGKVRPYV
14	BPSL1552 (121–140)	NHAGKVRPYVGAGINYTLFY
15	BPSL1552 (131–150)	GAGINYTLFYNNGLHAGGEG
16	BPSL1552 (141–160)	NNGLHAGGEGIGINNHSFGP
17	BPSL1552 (151–170)	IGINNHSFGPALQFGVDVQV
18	BPSL1552 (161–180)	ALQFGVDVQVTKKVFVNVDV
19	BPSL1552 (171–190)	TKKVFVNVDVKKIWMHTDAT
20	BPSL1552 (181–200)	KKIWMHTDATLGGQPLGRLN
21	BPSL1552 (191–214)	LGGQPLGRLNIDPLVVGVGVGMKF

### BpOmpW Induced T-Cell Proliferation in Human PMBCs

**Figure 6:**
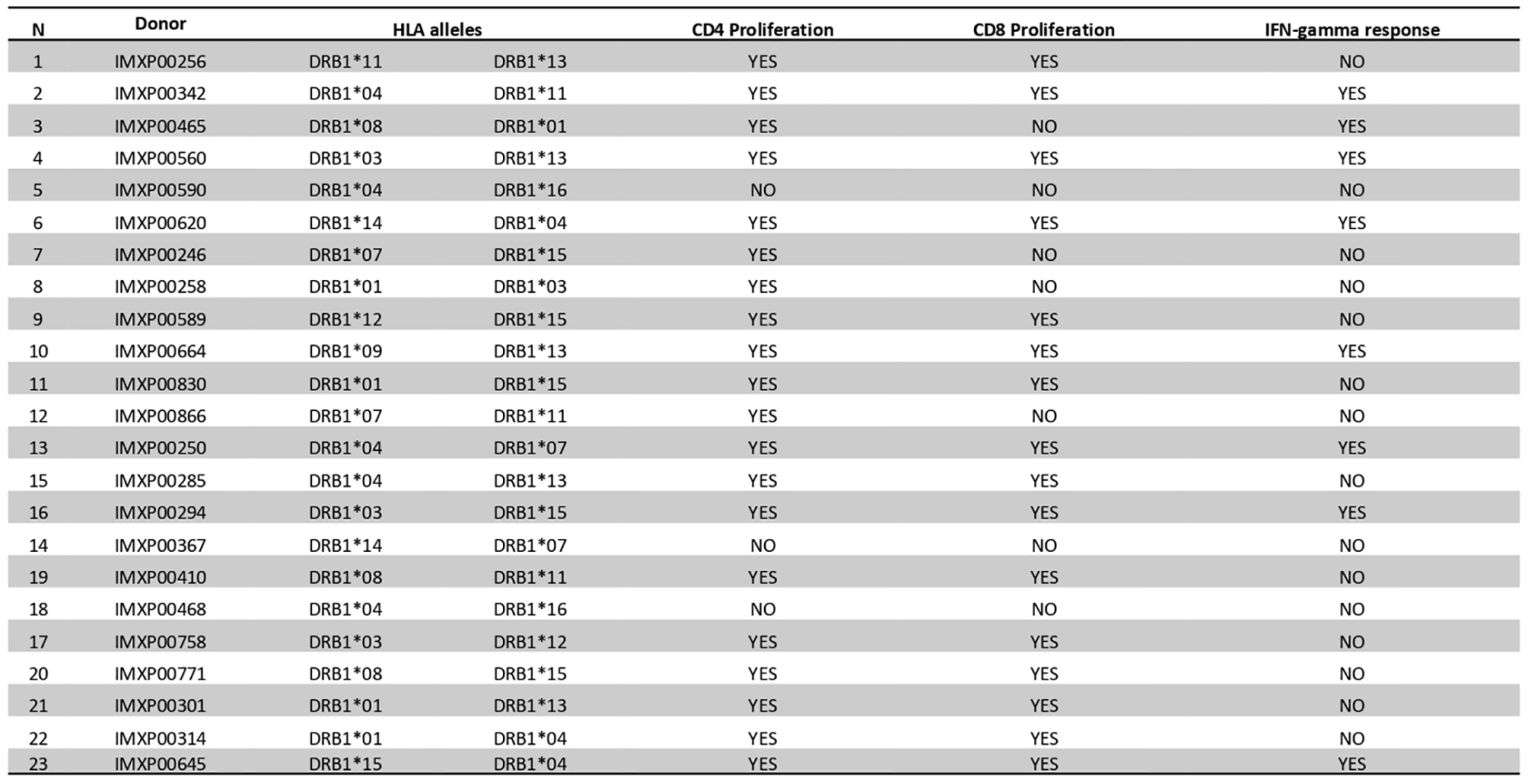
BpOmpW (homologue OmpW in *Burkholderia pseudomallei*) induced T-cell proliferation in human peripheral blood mononuclear cells (PBMCs). A table showing a qualitative description (YES/NO) based on the differential significance of CD4^+^ and CD8^+^ T-cell proliferation and the overall IFN-γ responses of 23 donor PBMCs in response to BpOmpW or BpOmpW+SAS and SAS control is included. The table also shows the human leukocyte antigen (HLA) alleles of each donor. *YES*/*NO* denote significant differences (*p* < 0.05) according to two-way ANOVA.

We examined the T-cell proliferation and IFN-γ responses of human PBMCs from healthy donors in response to BpOmpW exposure. We interrogated human PBMCs from 23 different donors with different HLA alleles (**Figure 6**) for CD4 and CD8 T-cell markers following exposure to BpOmpW with and without SAS adjuvant. T-cell proliferation in response to BpOmpW stimulation was confirmed in almost all donors (**Figure 6** and **Figures S12–S19**), as proliferating cells for the CD4 and CD8 populations were elevated in 87% and 70% of donors, respectively, following exposure to the antigen with or without SAS adjuvant relative to the untreated control group (**Figure 6** and **Figures S12–S19**). Eight donors out of 23 showed elevated IFN-γ responses in the presence of the antigen or when adjuvanted with respect to the control group (**Figure 6**).

### BpOmpW-Specific IgG Responses Occur in Melioidosis Survivors With Diabetes

**Figure 7:**
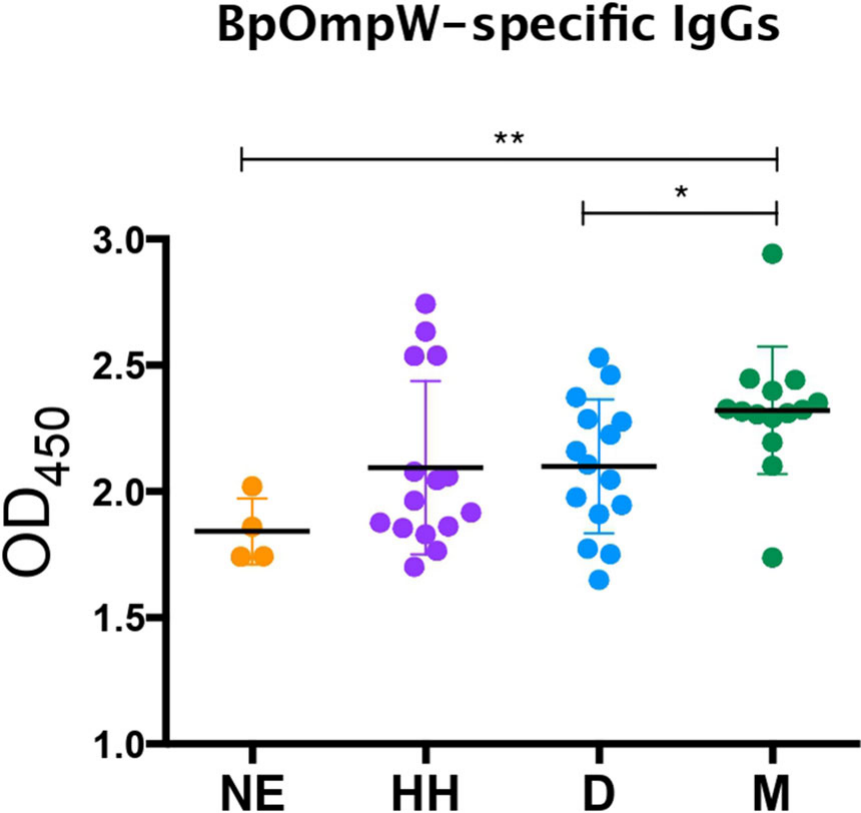
BpOmpW-specific immunoglobulin G (IgG) responses in plasma from melioidosis survivors. Detection by ELISA of the BpOmpW-specific IgGs in plasma from different cohorts: *NE*, non-endemic (*n* = 3); *HH*, healthy householders (*n* = 15); *D*, healthy diabetics (*n* = 15); *M*, melioidosis diabetic survivors (*n* = 15). *BpOmpW*, homologue OmpW in *Burkholderia pseudomallei Asterisks* mark significant differences according to two-tailed Student’s *t*-test. The levels of significance are represented as follows: *p < 0.05; **p < 0.01.

We have shown that BpOmpW stimulates strong serological responses in mice, both in non-insulin-resistant (10) and in insulin-resistant mice. In order to examine whether the antigen can stimulate a humoral response in melioidosis patients with diabetes, we examined the presence of BpOmpW-specific antibodies in sera from people with diabetes in Northeast Thailand who survived melioidosis infection (**Figure 7**). We showed that the melioidosis survivor cohort with diabetes (M) had significantly higher BpOmpW-specific IgG responses than the non-melioidosis cohort with diabetes (D; *p* = 0.0289) and the non-endemic healthy control (NE; *p* = 0.0023) groups, indicating that BpOmpW is specifically recognized following melioidosis exposure and is immunogenic in people with diabetes. No significant difference in the IgG levels was observed between the melioidosis cohort and a cohort of healthy household contacts of people with melioidosis (HH; *p* = 0.0537), most likely reflecting the endemic nature of melioidosis in Thailand.

## Discussion

Melioidosis is a neglected tropical disease caused by the obligate intracellular bacterium *Burkholderia pseudomallei*. The continued emergence of this pathogen throughout tropical and subtropical regions (29), together with the global increase of T2D (30), is a cause of major concern in these areas and has accelerated the search for new remedies to combat this disease (31). Although the protective immune response against *B. pseudomallei* is not fully understood, it is widely acknowledged that, due to the intracellular nature of *B. pseudomallei*, an effective vaccine should induce both T-cell and B-cell responses (32). Therefore, the elucidation of the protective cell-mediated immune responses of the BpOmpW antigen in preclinical studies is essential to advance the development of an efficacious human T-cell-inducing vaccine against melioidosis.

The strong humoral responses elicited by BpOmpW in our earlier study (10) were reproduced in this study in both non-insulin-resistant and insulin-resistant mice, confirming that the vaccine elicits potent specific antibody responses against BpOmpW. Splenocytes from BpOmpW-immunized mice re-exposed to BpOmpW showed substantial activation and differentiation relative to control mice, as shown, for example, in the expressions of CD25 and CD44 in both CD4 and CD8 T cells. T-cell activation also leads to CD45RB regulation, an essential marker that determines the fate of T cells from naive to effector/memory cells. The generation of effector T cells that will undergo development to memory cells is an essential correlate of protection in determining the efficacy of vaccines (33, 34). All effector subsets evaluated, i.e., effector CD4 and effector CD8, were increased in the BpOmpW-immunized group, indicating that the antigen produces a robust effector recall response that is likely to lead to a memory response, a hallmark of an efficacious vaccine. The early immune responses to control *B. pseudomallei* infection were predominantly mediated by IFN-γ in both CD4^+^ and CD8^+^ T cells and, as recently reported, in NK and DN cells (18, 19). In this study, all these IFN-γ-producing cell subsets were upregulated in the presence of the BpOmpW antigen, indicating that it elicits protective T-cell responses to combat the disease. Furthermore, the vaccine elicited a mixed Th response, which is in line with the reported dominant Th2 and Th17 responses at the initial stages of infection (28). The expression of the pro-inflammatory cytokine TNF is a typical murine host immune response to *B. pseudomallei* and was secreted in response to BpOmpW immunization. At the same time, the elevated numbers of regulatory T cells following BpOmpW immunization observed in non-insulin resistant mice are required to reduce the excessive pro-inflammatory cascade of cytokines by bacterial lipopolysaccharides (35). Finally, the fact that BpOmpW induced CD4^hi^ and CD8^hi^ populations suggested that both populations of CD4^hi^ and CD8^hi^ may change from activated CD45RB^lo^CD44^hi^ to naive-like CD45RB^veryhi^CD44^lo^ subsets in the BpOmpW group splenocytes.

C57BL/6J mice are widely used as a model for chronic melioidosis (24, 36). HFD-fed C57BL/6J mouse models have been previously used to study the immune responses impaired by DM in vaccine development (36–39). In agreement with previous reports, the mice gained weight and developed hyperglycemia and insulin resistance and showed the presence of lipid droplets in the liver within 12 weeks of HFD. DM alters the adaptive immune response to infections (36), including melioidosis (18). The immune response to BpOmpW in insulin-resistant mice was generally comparable to that observed in non-insulin-resistant mice, with some differences. BpOmpW re-exposure in insulin-resistant mice maintained an activated T-cell status and high IFN-γ recall responses from CD4, CD8, and NKT cells, which would be essential to protect people with diabetes from melioidosis. Non-insulin-resistant mice showed mixed Th1, Th2, and Th17 responses, in response to BpOmpW immunization in re-exposed splenocytes, in line with the Bcc-OmpW homolog examined previously (11). In contrast, insulin-resistant mice showed Th1 and Th17 responses with no apparent Th2 response following BpOmpW immunization. This is consistent with the fact that the C57BL/6J strain preferentially differentiates to the Th1 phenotype in response to HFD (40). Obesity, diabetes, and insulin resistance phenotypes produce pro-inflammatory cytokines that, in turn, are reported to downregulate the regulatory T cells required to prevent excessive inflammatory responses (41, 42). In this work, regulatory T cells remained comparable to those in the control in insulin-resistant mice, probably due to the stimulative effect of BpOmpW.

The strong recall response of all HLA transgenic mice tested to the complete BpOmpW antigen indicated that the T-cell responses identified in humans translate to IFN-γ responses. The T-cell proliferation observed in human PBMCs from different donors following BpOmpW exposure confirmed that the vaccine antigen is immunogenic for T-cell responses in humans. In contrast to other studies in which the identification of the candidate antigens was performed on the basis of their reactivity against patient antisera, we looked for BpOmpW-specific antibodies in plasma from different cohorts, including melioidosis survivors. The finding that BpOmpW also stimulated strong IgG responses in melioidosis survivors showed its potential to be a protective vaccine antigen against this disease, although the ultimate test will require human trials. Overall, the range of approaches used to elucidate whether the BpOmpW antigen elicits the necessary correlates of protection in humans strongly suggest that BpOmpW will elicit robust responses in humans and bring the vaccine closer to clinical trials.

## Data Availability Statement

The raw data supporting the conclusions of this article will be made available by the authors, without undue reservation.

## Ethics Statement

The studies involving human participants were reviewed and approved by the Ethics Committee of the Faculty of Tropical Medicine, Mahidol University (TMEC 12-014). The patients/participants provided written informed consent to participate in this study. The animal study was reviewed and approved by the University College Dublin Ethics Committee.

## Author Contributions

JT-C, LB, CQ, CR, DB, NC, MÓM, EM, and PR performed the experiments. JT-C, JhA, AB, DA, RB, and SMcC analyzed the data. JT-C, JA, AB, DA, RB, JhA, SG, and SMcC designed and supervised the experiments. SD and PR collected and provided the Thai clinical samples and data. JT-C, LB, CR, JA, AB, SD, SG, DA, and SMcC reviewed the manuscript. JT-C and SM wrote the manuscript. All authors contributed to the article and approved the submitted version.

## Conflict of Interest

Author MS was employed by company LIONEX Diagnostics and Therapeutics GmbH. Authors LB, JhA and SG were employed by Immunxperts company.

The remaining authors declare that the research was conducted in the absence of any commercial or financial relationships that could be construed as a potential conflict of interest.

## Publisher’s Note

All claims expressed in this article are solely those of the authors and do not necessarily represent those of their affiliated organizations, or those of the publisher, the editors and the reviewers. Any product that may be evaluated in this article, or claim that may be made by its manufacturer, is not guaranteed or endorsed by the publisher.
