# Supplementary material for: BpOmpW Antigen Stimulates the Necessary Protective T-Cell Responses Against Melioidosis
Source: Front Immunol. 2021 Dec 13;12:767359. doi: 10.3389/fimmu.2021.767359 (PMC8710444; doi:10.3389/fimmu.2021.767359)
Supplement: Supplementary file 1 [file DataSheet_1.pdf]

1. Experiment Overview

Purpose: The purpose of the experiment is to characterize T cell responses against BpOmpW vaccine antigen
Keywords: Splenocytes, vaccine, BpOmpW, antigen, T cell responses
Experiment Variables: Percentage of splenocytes exposed to BpOmpW antigen
Organization: Flow Cytometry Core at UCD Conway Institute. University College Dublin, Belfield, Dublin 4, Ireland. Eircode: D04 V1W8
Primary Contact: Julen Tomás Cortázar (julen.tomascortazar@ucd.ie)
Date: Non-insulin resistant experiment: Cytometry performed on February 8, 2020. Insulin resistant experiment: Cytometry performed on March 28, 2020.
Conclusions: The antigen induce the necessary T cell responses to combat melioidosis disease, even in insulin resistant mice
Quality control measures: FMOs were done for each marker. Splenocytes collected from SAS only adjuvanted mice as negative cells in order to provide a staining control.

2. Flow Sample/Specimen Details

Sample Material Description: Splenocytes from non- and insulin resistant C57Bl/6j mice stained with a panel antibody in 96wp. Mice were 6-8 weeks old in non-insulin resistance study. Mice were 6-8 weeks old fed by HFD for 16 weeks more in insulin resistance study. All mice were male immunized with SAS adjuvant alone or SAS + BpOmpW
Sample Characteristics: After lysing red blood cells from the mashed spleens, remaining splenocytes were stained for T cell markers and intracellular cytokines. One million of cells were plated in vitro and stimulated with BpOmpW antigen for 60 hours.
Sample Treatment Description: One million of splenocytes resuspended in 100ul PBS 1% FBS. Block Immunoglobulin FcReceptor. Purified Rat anti-Mouse BD FcBlockTM, CD16/CD32 (BD Pharmingen 553142) have been used to block nonspecific staining due to FcR. Cells were washed with PBS 1% FBS before using BD FcBlock. To block FcR, cells were incubated with one microliter BD FcBlock in 100ul PBS 1% FBS cell suspension for 5 minutes on ice in 96wp. Cells were washed and incubated with a panel of antibodies for 30 minutes on ice using 25ul PBS 1% FBS + 25ul Brilliant Stain Buffer from BD Horizon (563794). For intracellular staining, BD Cyofix/Cytoperm kit (BD 554714) was used. 100ul Cytofix/Cytoperm buffer were used for 20 minutes on ice to permeabilize and fix the cells in 96wp. Splenocytes were then intracellularly stained in 25ul Brilliant Stain Buffer + 25ul 1X Perm/Wash bufer in 96wp. Cells were washed twice with 200ul 1X Perm/Wash buffer and leave overnight in this buffer. Finally, next day, splenocytes were resuspended in PBS 1% FBS for flow cytometry analysis. All centrifugations were done at 300g for 5 min.

Fluorescence Reagent Description: Each sample has been stained and assigned to a Laser/channel according to the following table.

LASER/CHANNEL	Fluorophore-Antibody.
B525-FITC	FITC Rat Anti-Mouse CD8a (BD Pharmingen 553030)
B690-PC5.5	BB700 Rat Anti-Mouse TNF (BD Horizon 566510)
Y585-PE	PE Armenian Hamster anti-Mouse IL-9 (BD Pharmingen 561463)
Y763-PC7	PE-Cy7 Rat Anti-Mouse CD25 (BD Pharmingen 552880)
R660-APC	Alexa Fluor 647 Rat anti-Mouse Foxp3 (BD Pharmingen 560401)
R712-APCA700	APC-R700 Rat Anti-Mouse CD44 (BD Horizon 565480)
R763-APCA750	APC Cy7 Rat Anti-Mouse CD3 (BD Pharmingen 560690)
V450-PB	BV421 Rat Anti-Mouse IL-2 (BD Pharmingen 562969)
V525-KrO	BV510 Rat Anti-Mouse CD45RB (BD OptiBuild 740107)
V610	BV605 Rat Anti-Mouse IL-4 (BD Horizon 564007)
V660	BV650 Rat Anti-Mouse IFN-y (BD Horizon 563854)
V763	BV786 Rat Anti-Mouse IL-17A (BD Horizon 564171
NUV405	BUV395 Rat Anti Mouse CD4 (BD Horizon 563790)
NUV525	BUV563 Hamster Anti-Mouse CD49b (BD OptiBuild 741280)
IR885	ViaKrome 808 Fixable Viability Dye (Beckman Coulter C36628)

3. Instrument Details

Instrument Manufacturer: Beckman Coulter: https://www.beckmancoulter.com/
Instrument Model:
CytoFlex LX. Model No B90883.
Serial Number BA34050.

Instrument configuration and settings:

Flow Cell and Fluidics: The instrument has not been altered; fixed-alignment cuvette flow cell.

Light Sources: The instrument has not been altered.

Optical Filters: The instrument has not been altered.

Optical detectors: The instrument has not been altered.

The following figure shows the filter and detector configuration:

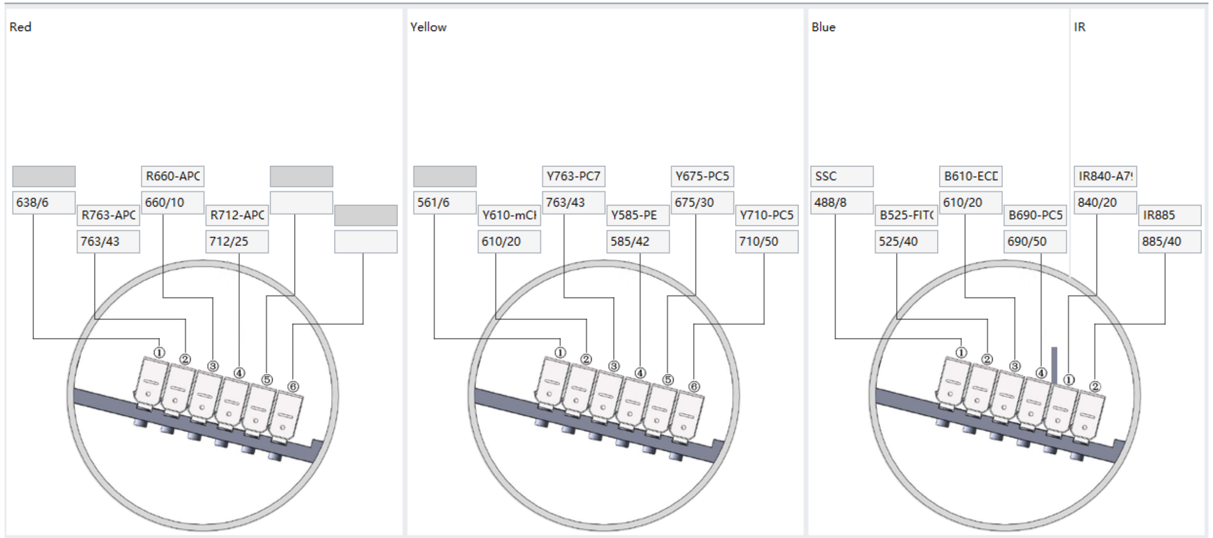

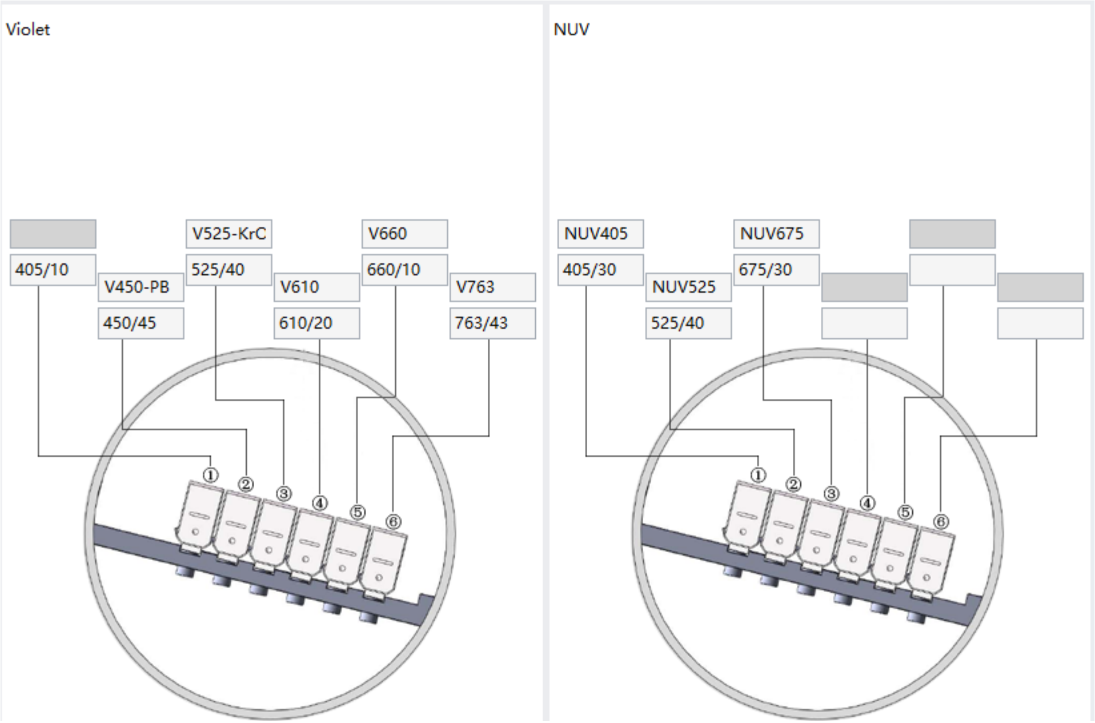

4. Data Analysis Details

List-mode Data Files: FCS data files can be obtained by contacting Dr Julen Tomás Cortázar or Assoc Prof. Siobhán McClean.
Data Transformation Details:
Purpose of Data Transformation: Visualization and gating.
Data Transformation Description: Cytexpert software
Compensation Description:

Autofl.	Channel	B525-FITC	B610-ECD	B690-PC5.5	Y585-PE	Y610- mCHERRY	Y675 -PC5	Y710- PC5.5	Y763 -PC7	R660-APC	R712-APCA700	R763-APC750	V450- PB	V525-Kr0	V610	V660	V763	NUV405	NUV525	NUV675	IR840-A7...	IR885
1,19	B525-FITC		0,00	3,33	2,06	0,00	0,00	0,00	51,95	0,00	0,07	0,00	74,58	1,24	0,00	0,00	0,04	0,00	0,00	0,00	0,00	0,00
0,00	B610-ECD	0,00		0,00	0,00	0,00	0,00	0,00	0,00	0,00	0,00	0,00	0,00	0,00	0,00	0,00	0,00	0,00	0,00	0,00	0,00	0,00
0,33	B690-PC5.5	0,00	0,00		7,55	0,00	0,00	0,00	88,12	0,92	3,25	0,39	0,00	0,01	0,00	1,97	0,23	0,00	0,00	0,00	0,00	1,40
0,63	Y585-PE	0,00	0,00	0,00		0,00	0,00	0,00	2,22	0,04	0,03	0,14	0,00	0,02	11,96	0,13	0,05	0,00	0,00	0,00	0,00	0,00
0,00	Y610- mCHERRY	0,00	0,00	0,00	0,00		0,00	0,00	0,00	0,00	0,00	0,00	0,00	0,00	0,00	0,00	0,00	0,00	0,00	0,00	0,00	0,00
0,00	Y675 -PC5	0,00	0,00	0,00	0,00	0,00		0,00	0,00	0,00	0,00	0,00	0,00	0,00	0,00	0,00	0,00	0,00	0,00	0,00	0,00	0,00
0,00	Y710- PC5.5	0,00	0,00	0,00	0,00	0,00	0,00		0,00	0,00	0,00	0,00	0,00	0,00	0,00	0,00	0,00	0,00	0,00	0,00	0,00	0,00
0,94	Y763 -PC7	0,01	0,00	0,03	3,40	0,00	0,00	0,00		0,13	0,21	1,11	0,02	0,01	0,22	0,15	0,00	0,00	0,00	0,00	0,00	0,00
0,32	R660-APC	0,00	0,00	3,61	0,10	0,00	0,00	0,00	2,77		9,96	0,00	0,00	0,00	0,13	29,20	0,21	0,03	0,00	0,00	0,00	0,00
0,11	R712-APCA700	0,00	0,00	15,61	0,05	0,00	0,00	0,00	3,60	20,31		7,13	0,00	0,00	0,04	7,44	0,33	0,00	0,00	0,00	0,00	12,22
1,39	R763-APC750	0,00	0,00	6,52	0,01	0,00	0,00	0,00	0,00	3,68	38,28		0,00	0,00	0,00	2,79	7,09	0,09	0,00	0,00	0,00	23,35
15,33	V450- PB	0,00	0,00	0,00	0,00	0,00	0,00	0,00	0,00	0,40	0,37	0,00		17,42	5,53	14,95	25,86	0,00	0,00	0,00	0,00	0,00
5,96	V525-Kr0	1,51	0,00	0,00	0,00	0,00	0,00	0,00	0,00	0,22	0,22	0,00	9,78		0,00	1,79	1,65	0,80	0,00	0,00	0,00	0,00
1,95	V610	1,66	0,00	0,97	9,61	0,00	0,00	0,00	2,42	0,11	0,13	0,00	0,23	25,11		39,69	0,00	0,00	0,00	0,00	0,00	0,00
0,22	V660	0,00	0,00	2,02	1,12	0,00	0,00	0,00	0,23	0,19	0,43	1,07	0,03	6,20	36,60		0,19	0,60	0,00	0,00	0,00	0,00
0,34	V763	0,01	0,00	9,88	0,24	0,00	0,00	0,00	0,00	0,12	5,57	16,88	0,01	2,16	11,67	28,74		0,06	0,00	0,00	0,00	9,48
6,07	NUV405	0,00	0,00	0,00	0,00	0,00	0,00	0,00	39,97	1,09	0,79	0,00	17,25	0,35	0,00	0,00	0,00		0,00	0,00	0,00	0,00
2,47	NUV525	16,57	0,00	0,00	0,00	0,00	0,00	0,00	0,00	0,77	0,60	0,00	5,72	69,21	0,00	1,17	0,00	8,96		0,00	0,00	0,00
0,00	NUV675	0,00	0,00	0,00	0,00	0,00	0,00	0,00	0,00	0,00	0,00	0,00	0,00	0,00	0,00	0,00	0,00	0,00	0,00		0,00	0,00
0,00	IR840-A7...	0,00	0,00	0,00	0,00	0,00	0,00	0,00	18,11	0,00	0,00	1,88	0,00	0,00	0,00	0,00	1,42	0,00	0,00	0,00		0,00
0,00	IR885	0,00	0,00	0,00	0,00	0,00	0,0-0	0,00	9,14	0,00	0,00	1,09	0,00	0,00	0,00	0,00	0,75	0,00	0,00	0,00	0,00	
