# Supplementary material for: BpOmpW Antigen Stimulates the Necessary Protective T-Cell Responses Against Melioidosis
Source: Front Immunol. 2021 Dec 13;12:767359. doi: 10.3389/fimmu.2021.767359 (PMC8710444; doi:10.3389/fimmu.2021.767359)
Supplement: Supplementary file 2 [file DataSheet_2.pdf]

Figure S1

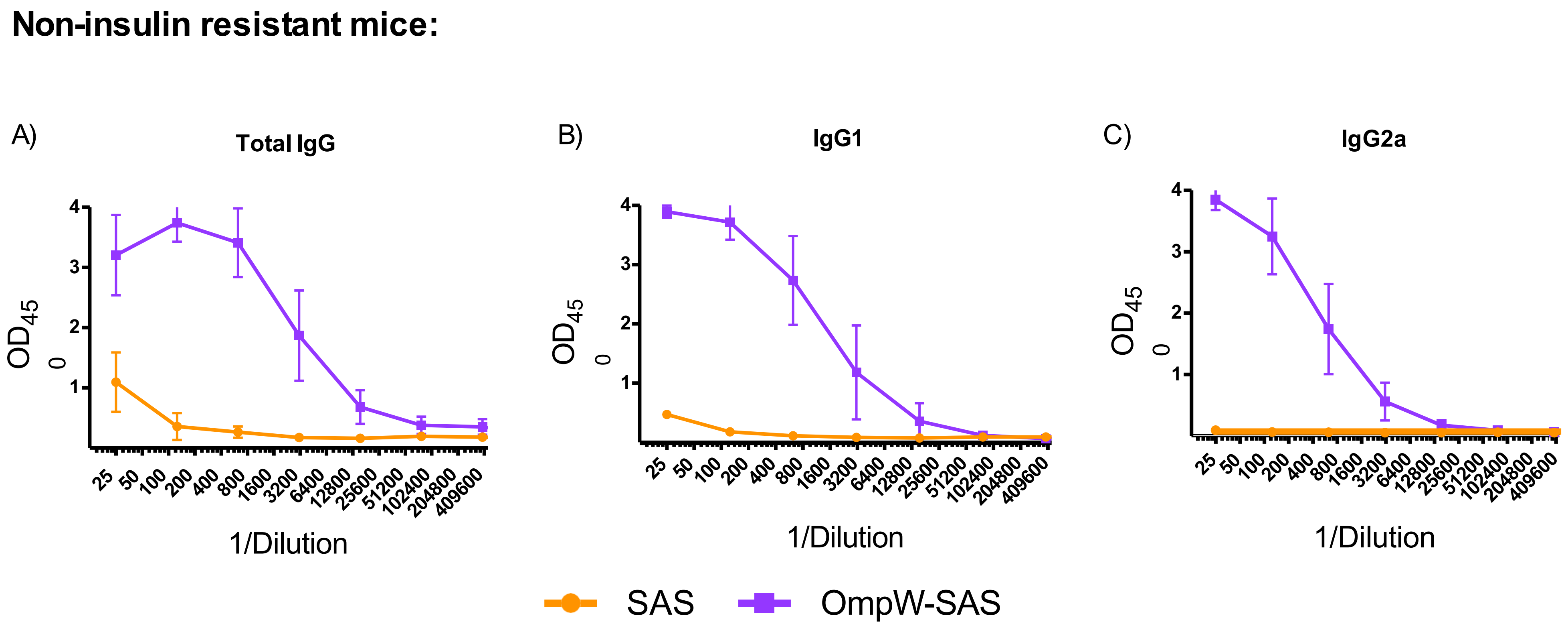
Non-insulin resistant mice:
A)
Total IgG
B)
IgG1
C)
IgG2a
OD450
1/Dilution
25 50 100 200 400 800 1600 3200 6400 12800 25600 51200 102400 204800 409600
SAS
OmpW-SAS

Figure S2

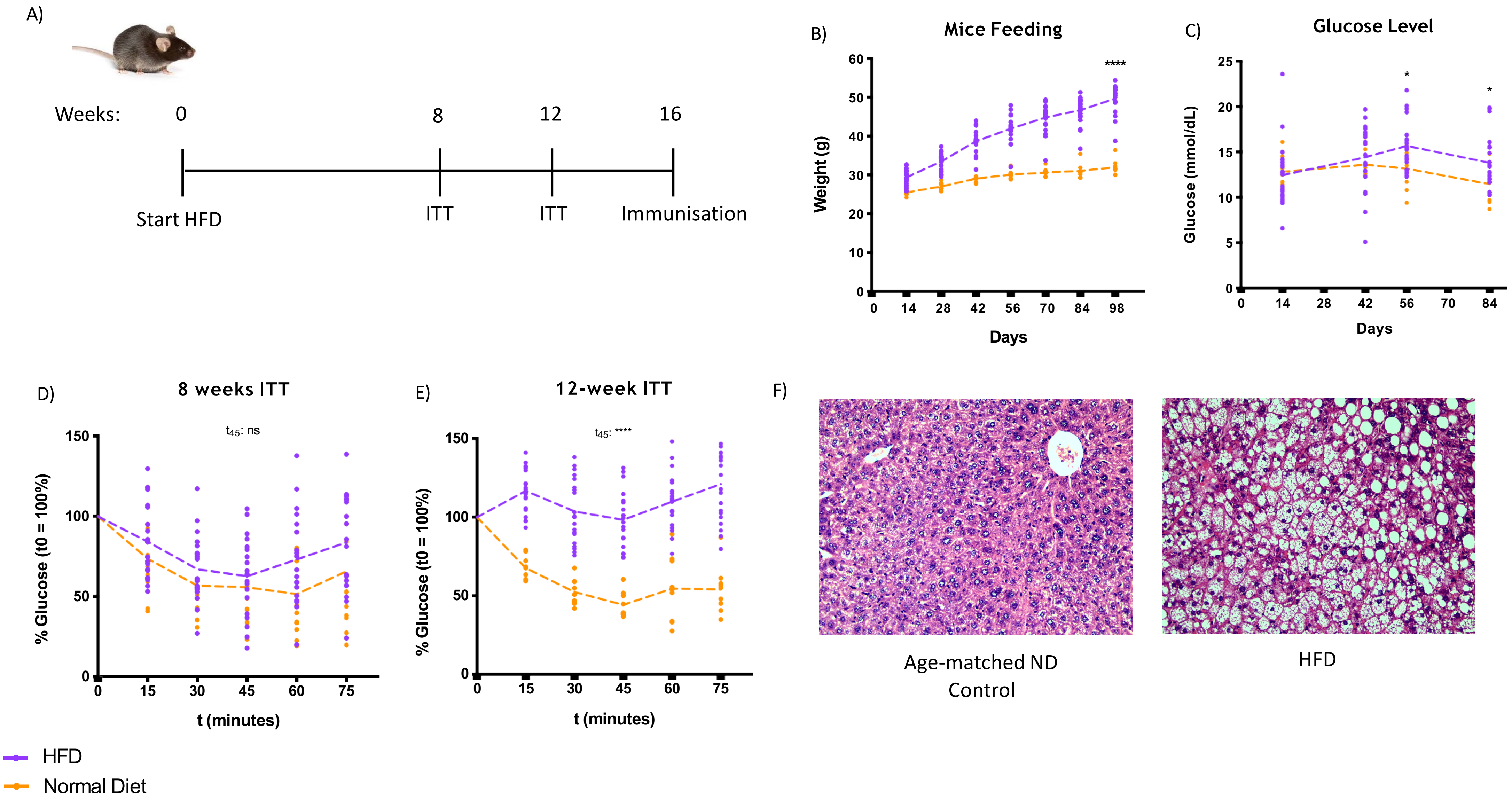
A)
Weeks: 0 8 12 16
Start HFD
ITT
ITT
Immunisation
B)
Mice Feeding
Weight (g)
Days
****
C)
Glucose Level
Glucose (mmol/dL)
Days
*
*
D)
8 weeks ITT
t_{45}: ns
% Glucose (t0 = 100%)
t (minutes)
E)
12-week ITT
t_{45}: ****
% Glucose (t0 = 100%)
t (minutes)
F)
Age-matched ND
Control
HFD
HFD
Normal Diet

Figure S3

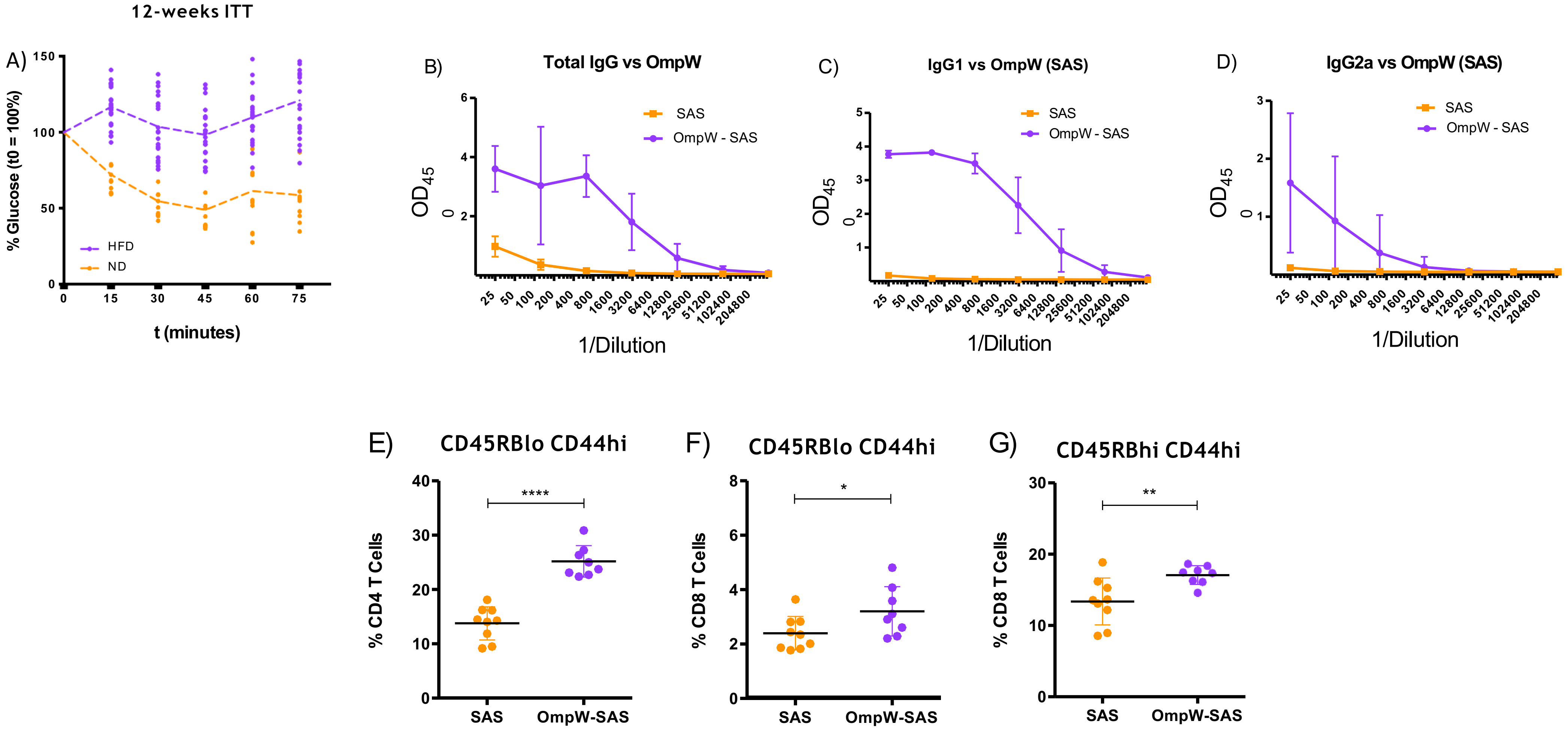

A) 12-weeks Insulin Tolerant Test (ITT) of mice in the insulin resistant mice. B-D) Antibody responses against BpOmpW in the insulin resistant mice. B) Total IgG against BpOmpW C) IgG1 responses against BpOmpW D) IgG2a responses against BpOmpW. E-G) Effector CD45RBlow CD44high CD4 T cells (E), effectors CD45RBlow CD44hi (F) and CD45RBhi CD44hi in CD8 T cells after BpOMpW stimulation in the insulin resistant mice.

Figure S4

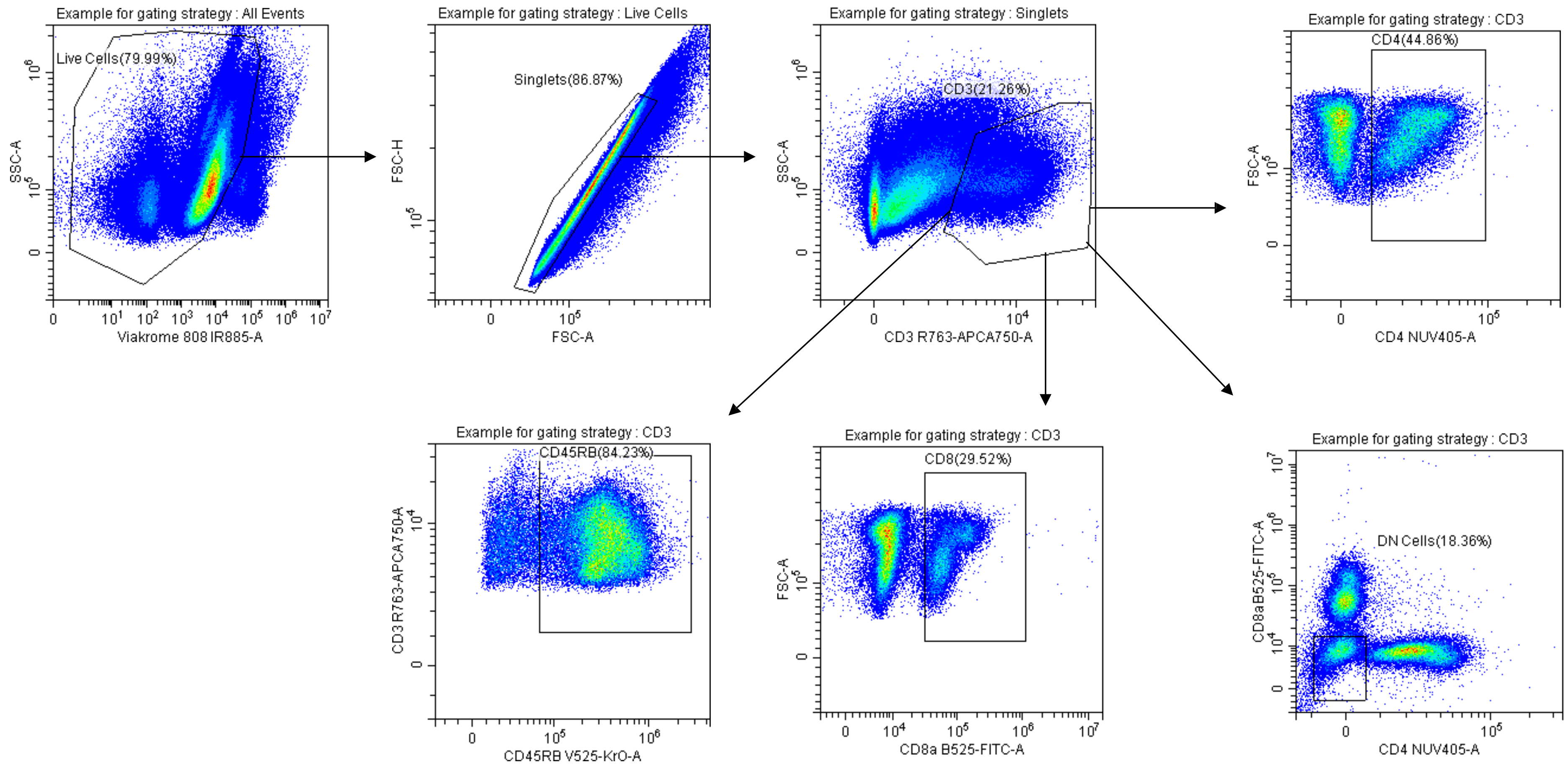

Gating Strategy for Live Cells, Singlets, CD3, CD4, CD8 and DN cells

Figure S5

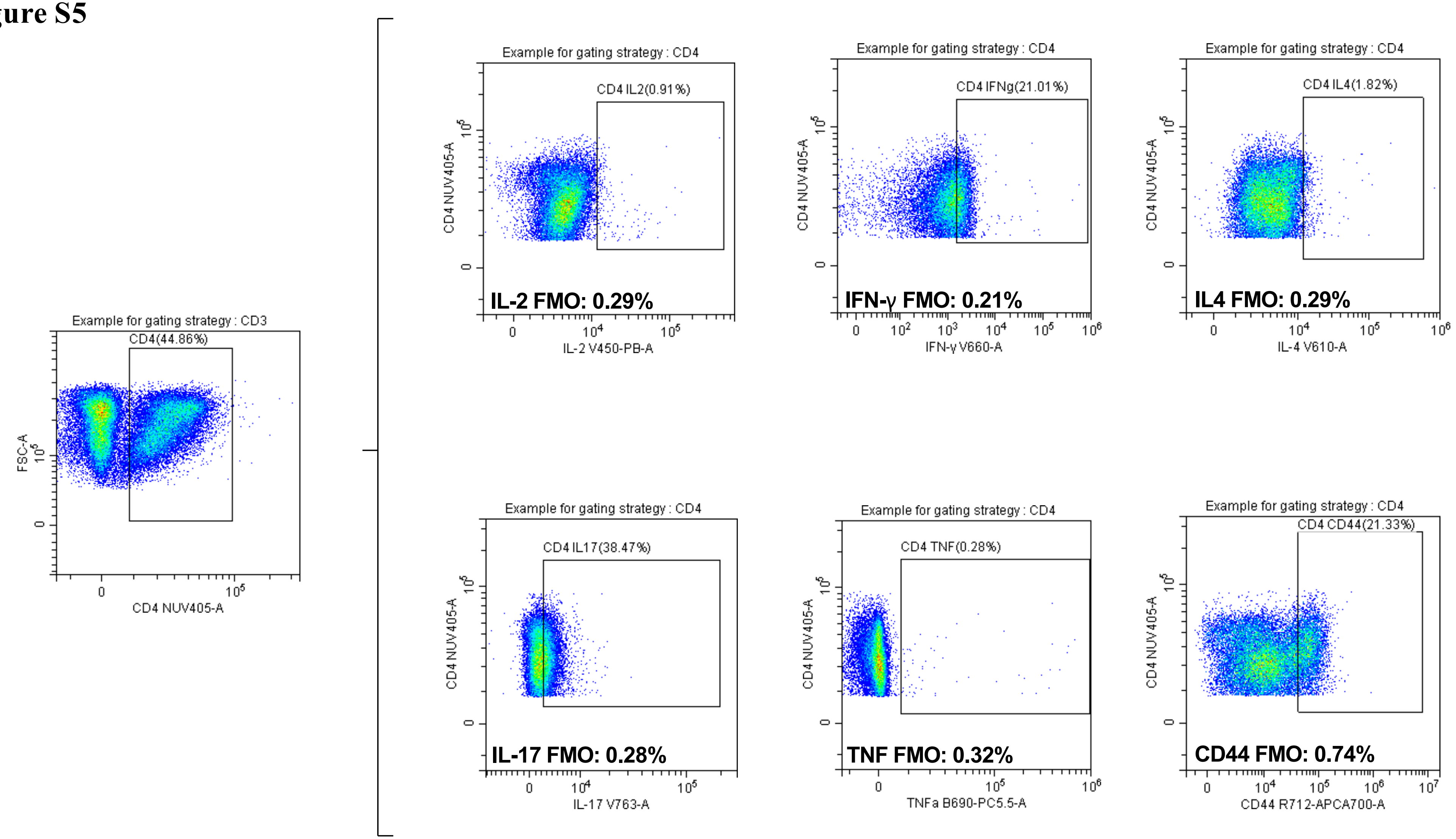

Gating strategy for CD4, the activation marker CD44 in CD4 T cells and secreted cytokines such as IL2, IFNy, IL4, IL17, TNF with their corresponding FMOs for each gate.

Figure S6

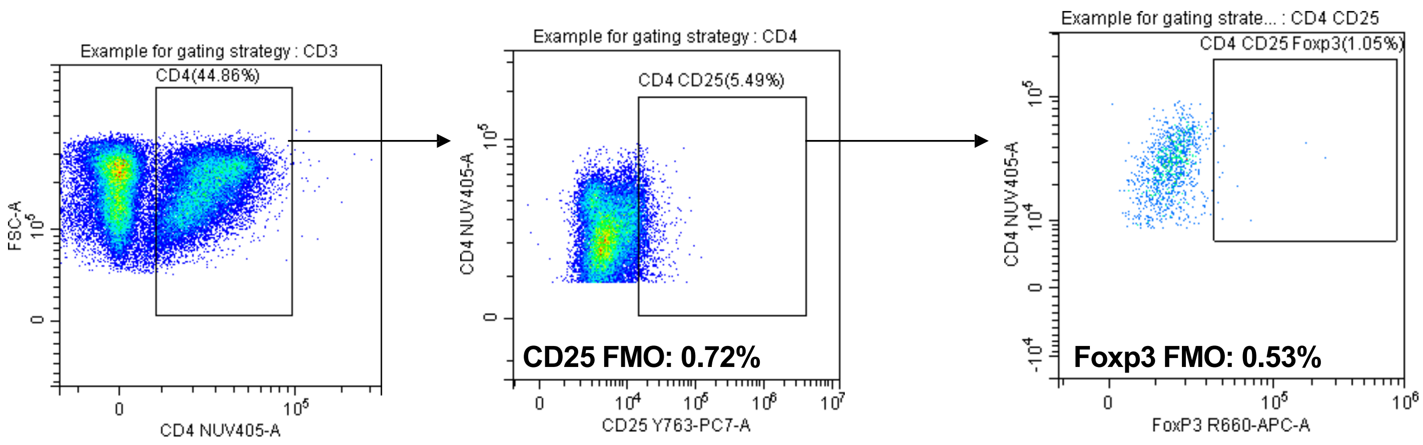

Gating strategy for CD4, the activation marker CD25 in CD4 T cells and the regulatory T cells CD4+ CD25+ Foxp3+

Figure S7

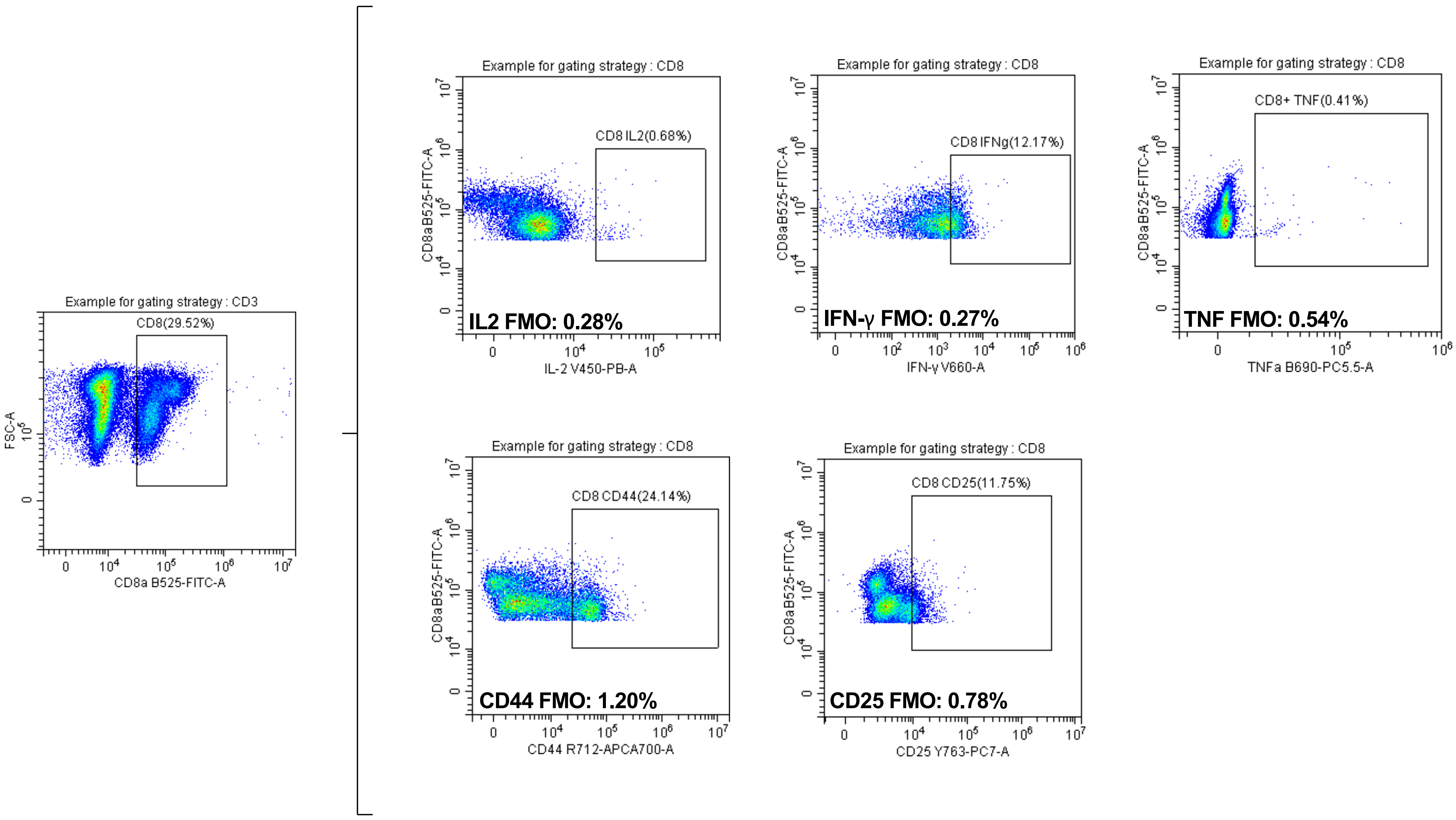

Gating strategy for CD8, the activation markers CD44 and CD25 in CD8 T cells and secreted cytokines such as IL2, IFNy and TNF with their corresponding FMOs for each gate.

Figure S8

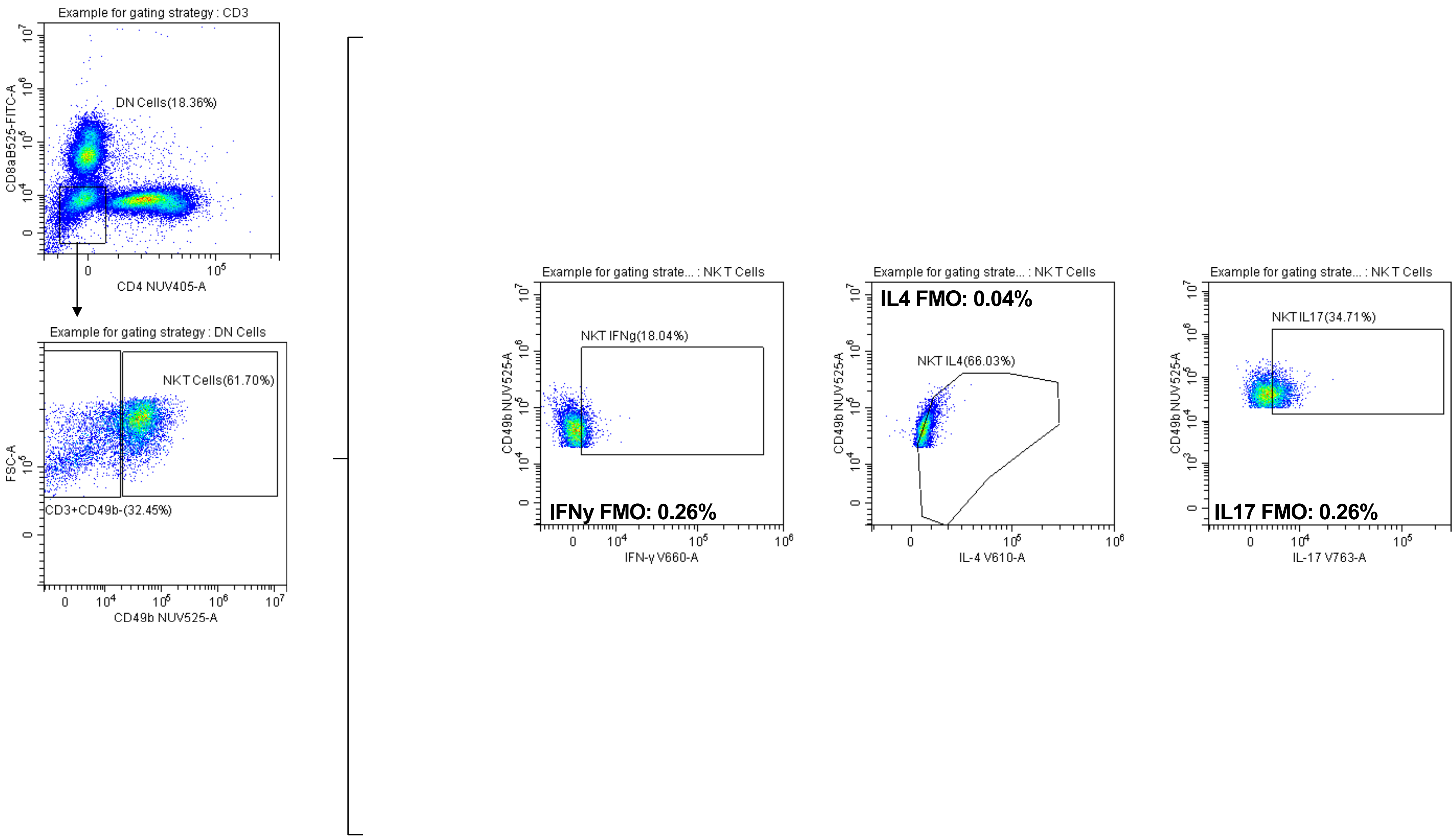

Example for gating strategy : CD3
DN Cells(18.36%)
CD8aB525-FITC-A
CD4 NUV405-A
Example for gating strategy : DN Cells
NKT Cells(61.70%)
CD3+CD49b-(32.45%)
FSC-A
CD49b NUV525-A
Example for gating strate... : NKT Cells
NKT IFNg(18.04%)
IFNy FMO: 0.26%
CD49b NUV525-A
IFN-γ V660-A
Example for gating strate... : NKT Cells
IL4 FMO: 0.04%
NKT IL4(66.03%)
CD49b NUV525-A
IL-4 V610-A
Example for gating strate... : NKT Cells
NKT IL17(34.71%)
IL17 FMO: 0.26%
CD49b NUV525-A
IL-17 V763-A

Figure S9

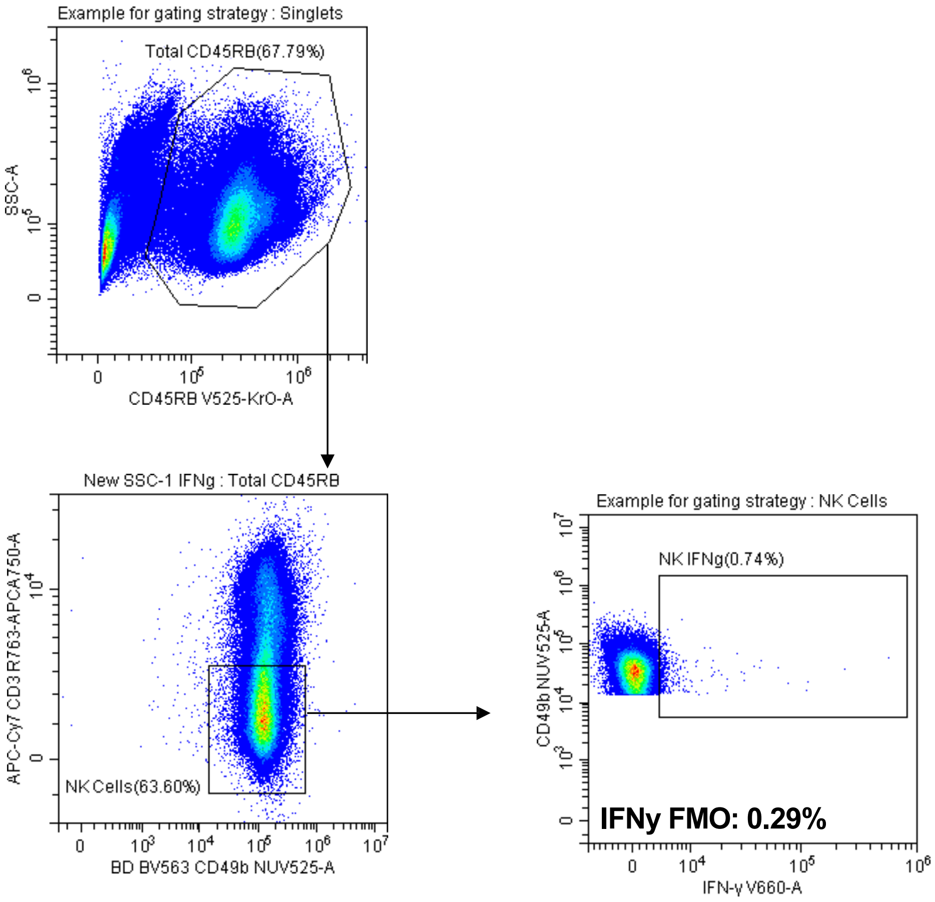

Gating strategy for NK cells and secreted IFNy with its corresponding FMO.

Figure S10

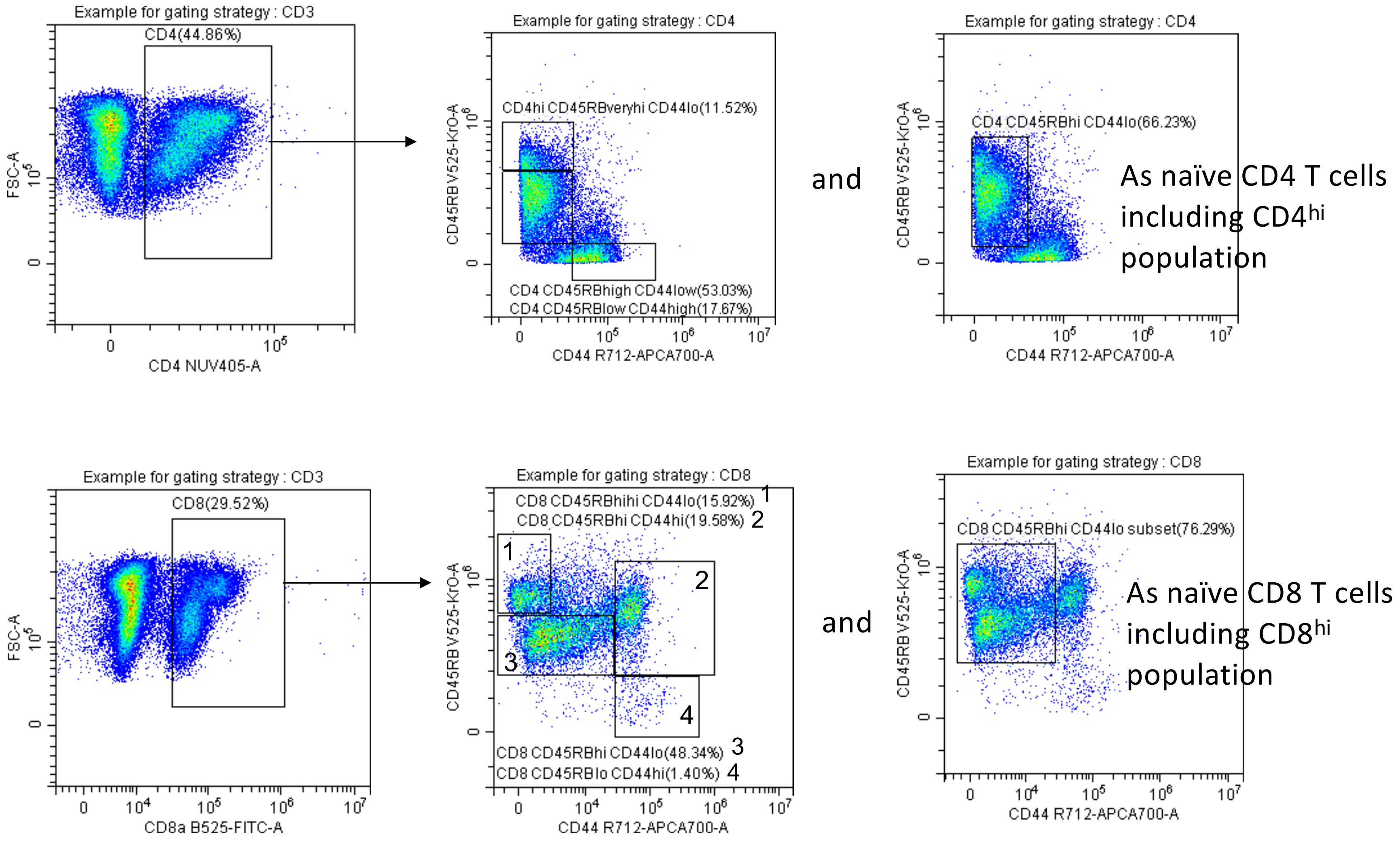

Gating strategy for CD4 and CD8 naïve and effector T cells classified by CD45RB and CD44 expression levels

Figure S11

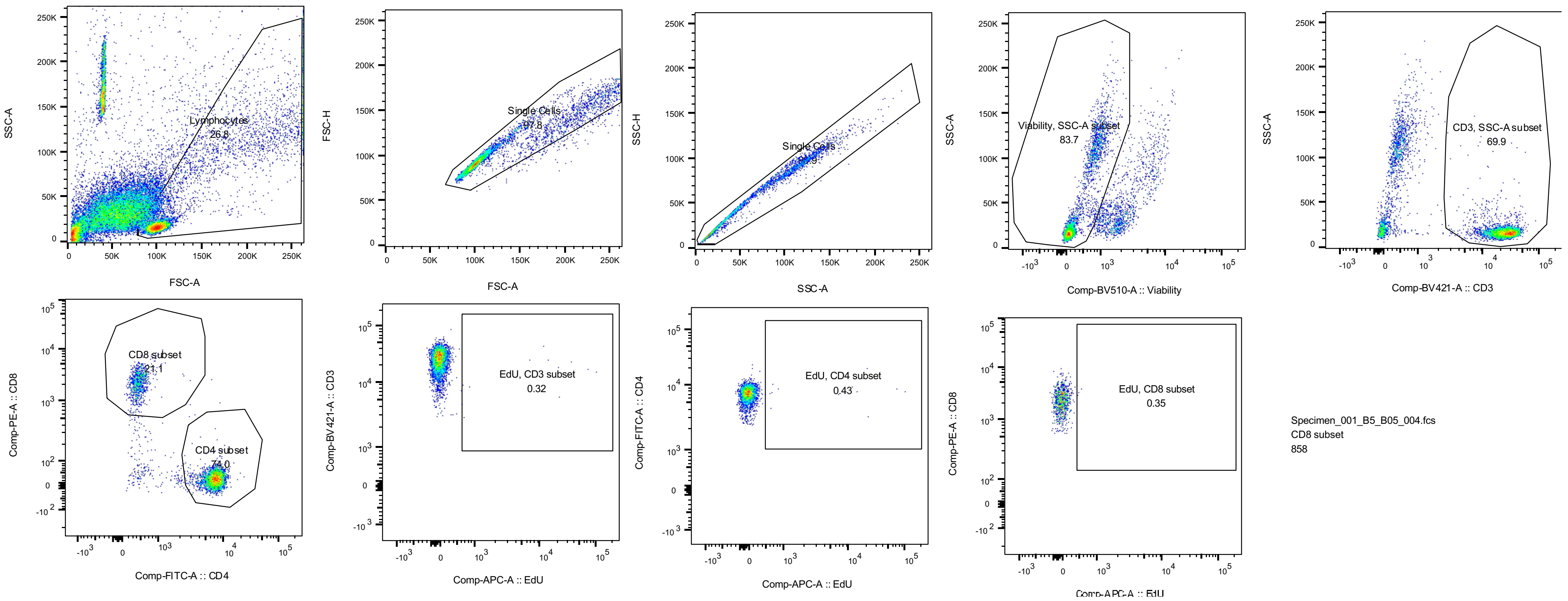

Lymphocytes
26.8
SSC-A
FSC-A
Single Cells
97.8
FSC-H
FSC-A
SSC-H
Single Cells
SSC-A
Viability, SSC-A subset
83.7
SSC-A
Comp-BV510-A :: Viability
CD3, SSC-A subset
69.9
SSC-A
Comp-BV421-A :: CD3
CD8 subset
21.1
CD4 subset
74.0
Comp-PE-A :: CD8
Comp-FITC-A :: CD4
EdU, CD3 subset
0.32
Comp-BV421-A :: CD3
Comp-APC-A :: EdU
EdU, CD4 subset
0.43
Comp-FITC-A :: CD4
Comp-APC-A :: EdU
EdU, CD8 subset
0.35
Comp-PE-A :: CD8
Comp-APC-A :: EdU
Specimen_001_B5_B05_004.fcs
CD8 subset
858

Figure S12

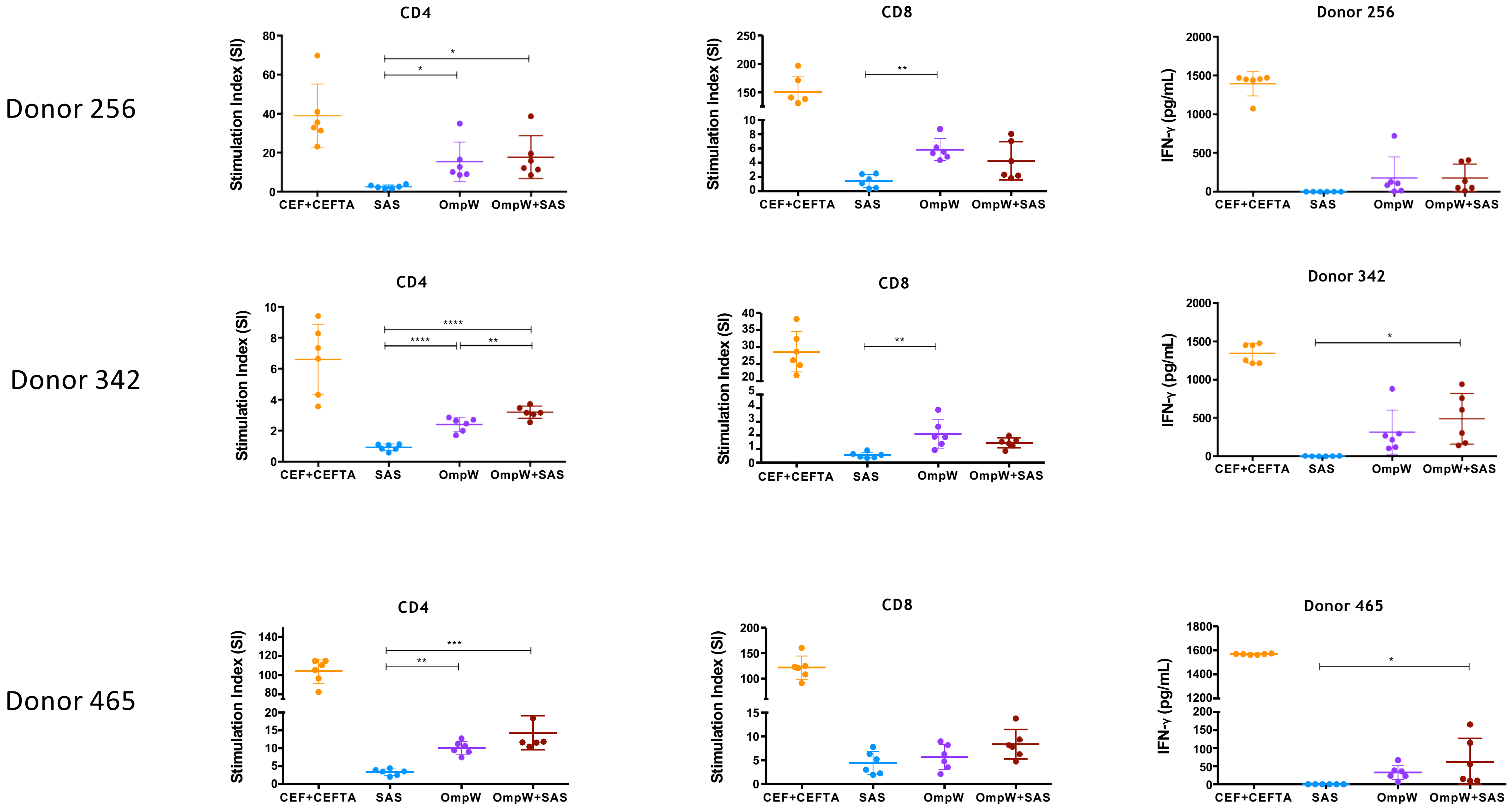

Donor 256
Donor 342
Donor 465
CD4
CD8
Donor 256
Donor 342
Donor 465
Stimulation Index (SI)
IFN-γ (pg/mL)
CEF+CEFTA
SAS
OmpW
OmpW+SAS

Figure S13

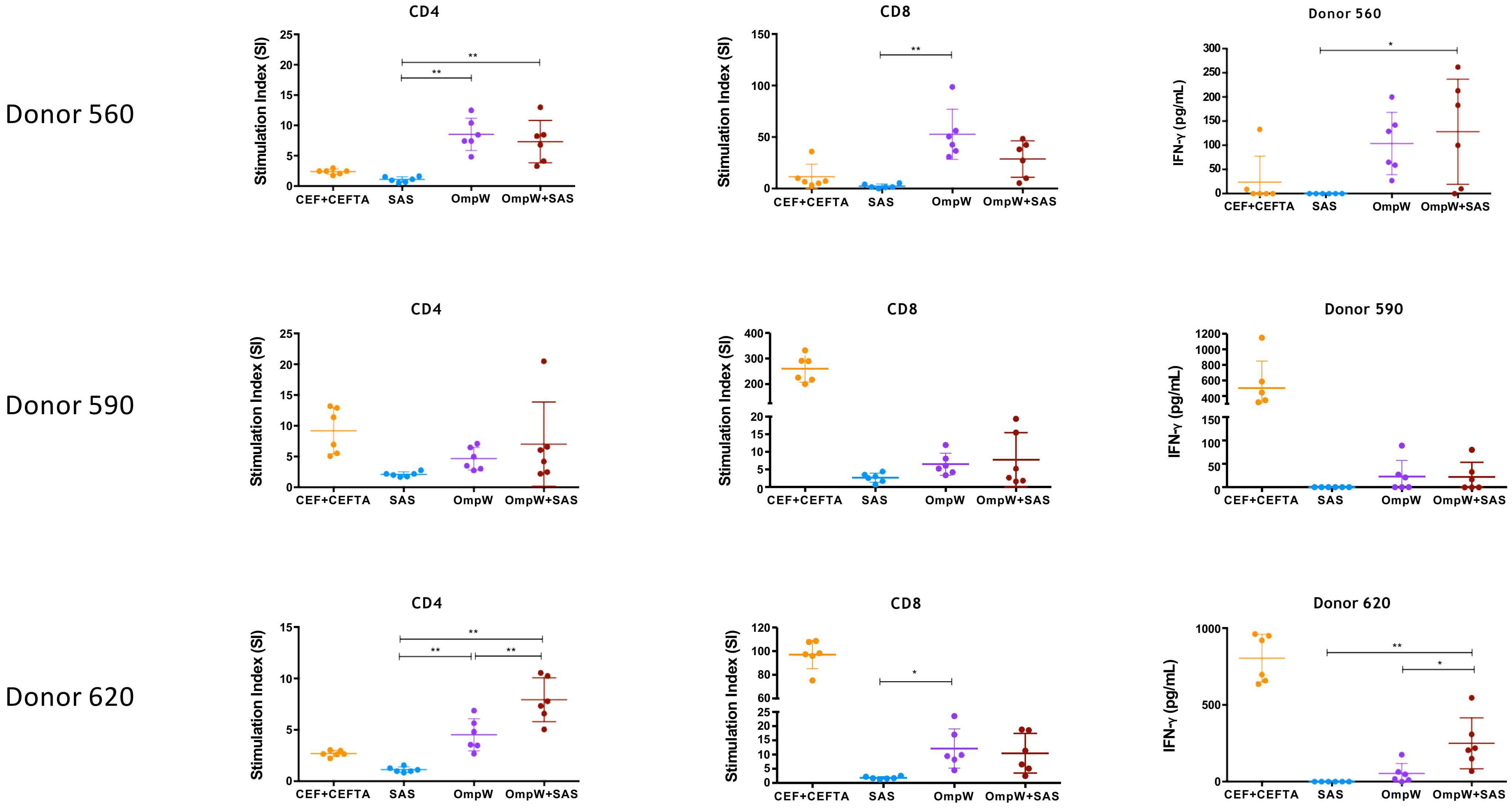
Donor 560
Donor 590
Donor 620
CD4
CD8
Donor 560
Donor 590
Donor 620
Stimulation Index (SI)
IFN-γ (pg/mL)
CEF+CEFTA
SAS
OmpW
OmpW+SAS
*
**

Figure S14

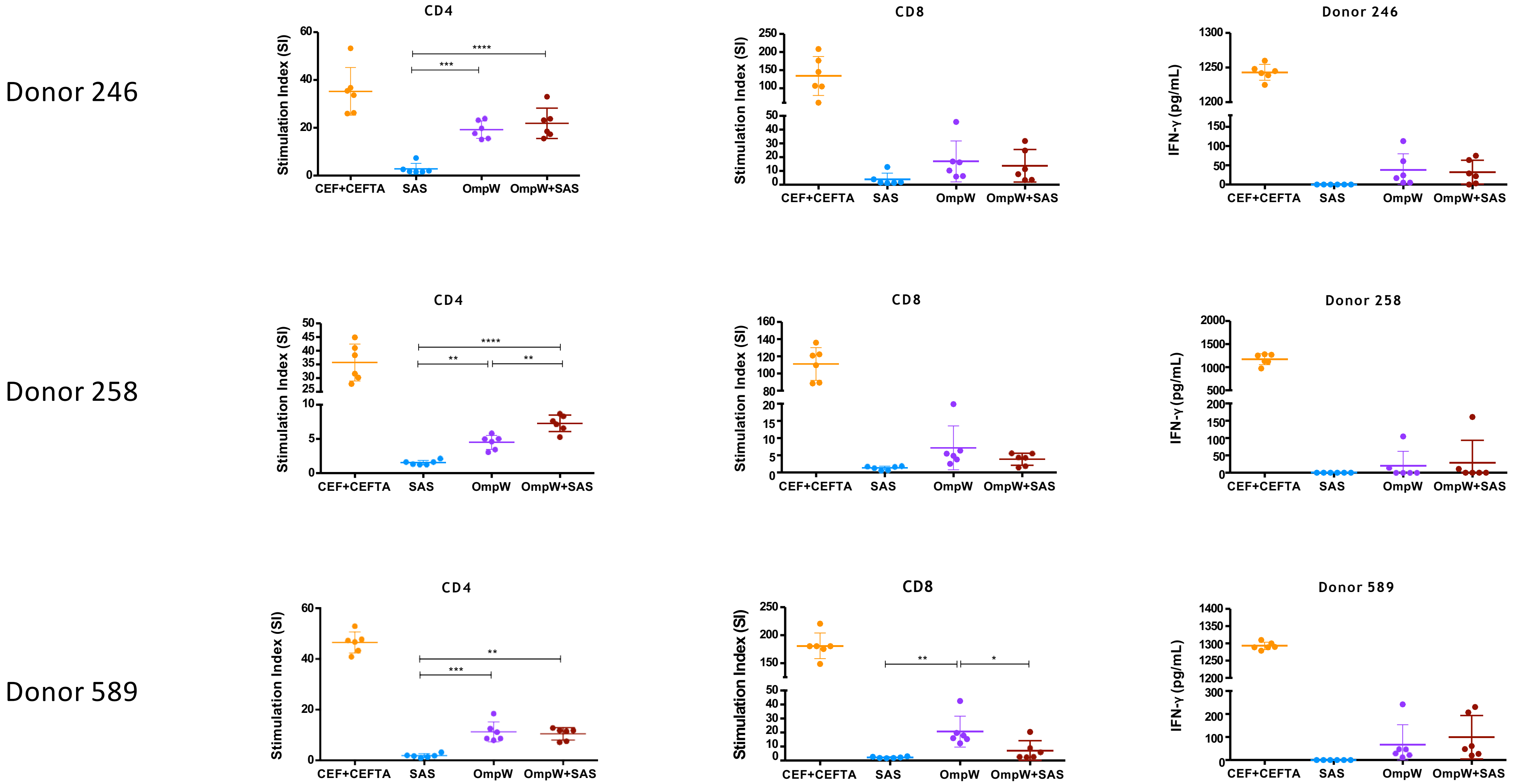
Donor 246
Donor 258
Donor 589
CD4
CD8
Donor 246
Donor 258
Donor 589
Stimulation Index (SI)
IFN-γ (pg/mL)
CEF+CEFTA
SAS
OmpW
OmpW+SAS
****
***
**
*

Figure S15

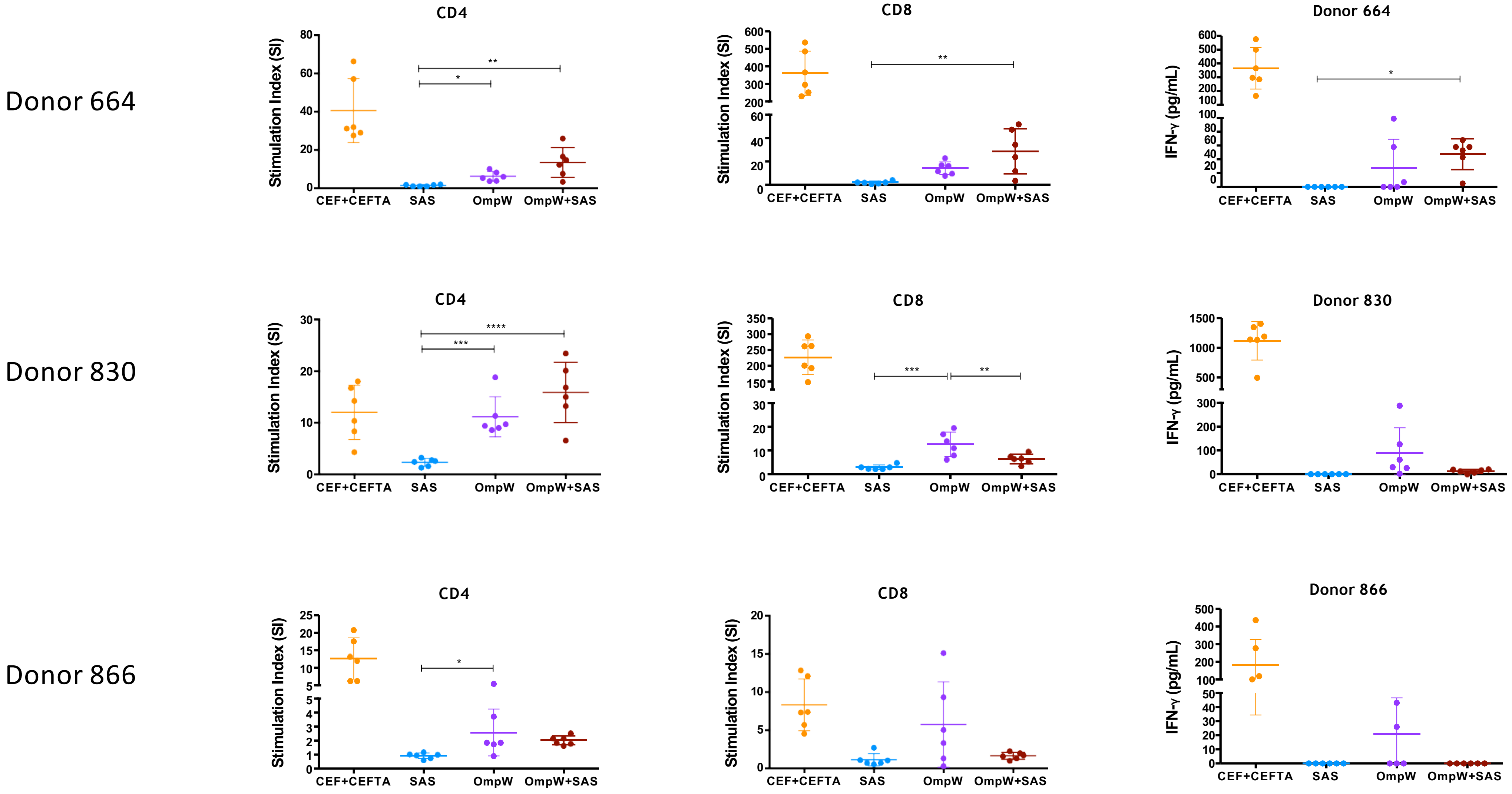
Donor 664
Donor 830
Donor 866
CD4
CD8
Donor 664
Donor 830
Donor 866
Stimulation Index (SI)
IFN-γ (pg/mL)
CEF+CEFTA
SAS
OmpW
OmpW+SAS

Figure S16

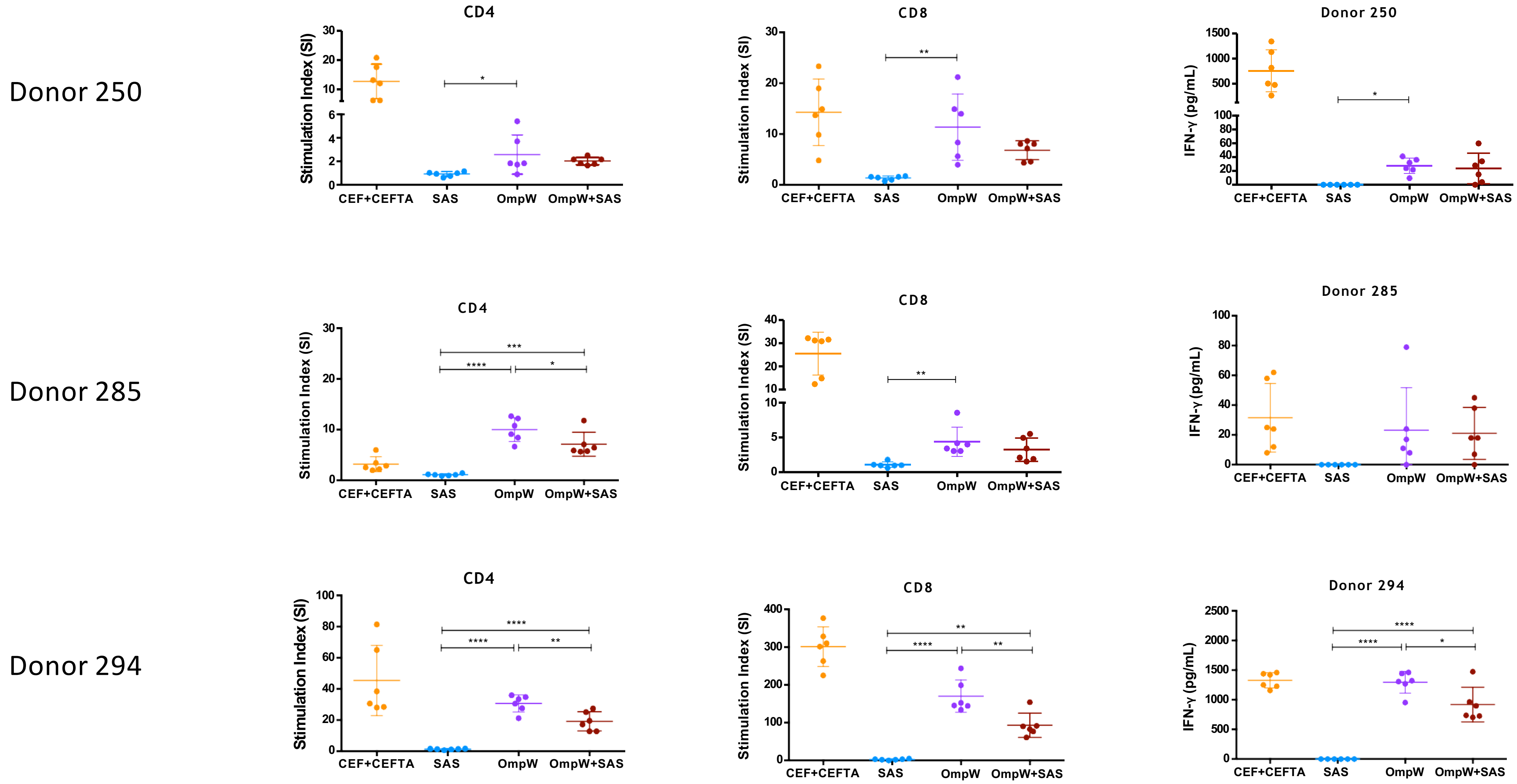
Donor 250
Donor 285
Donor 294
CD4
CD8
Donor 250
Donor 285
Donor 294
Stimulation Index (SI)
IFN-γ (pg/mL)
CEF+CEFTA
SAS
OmpW
OmpW+SAS

Figure S17

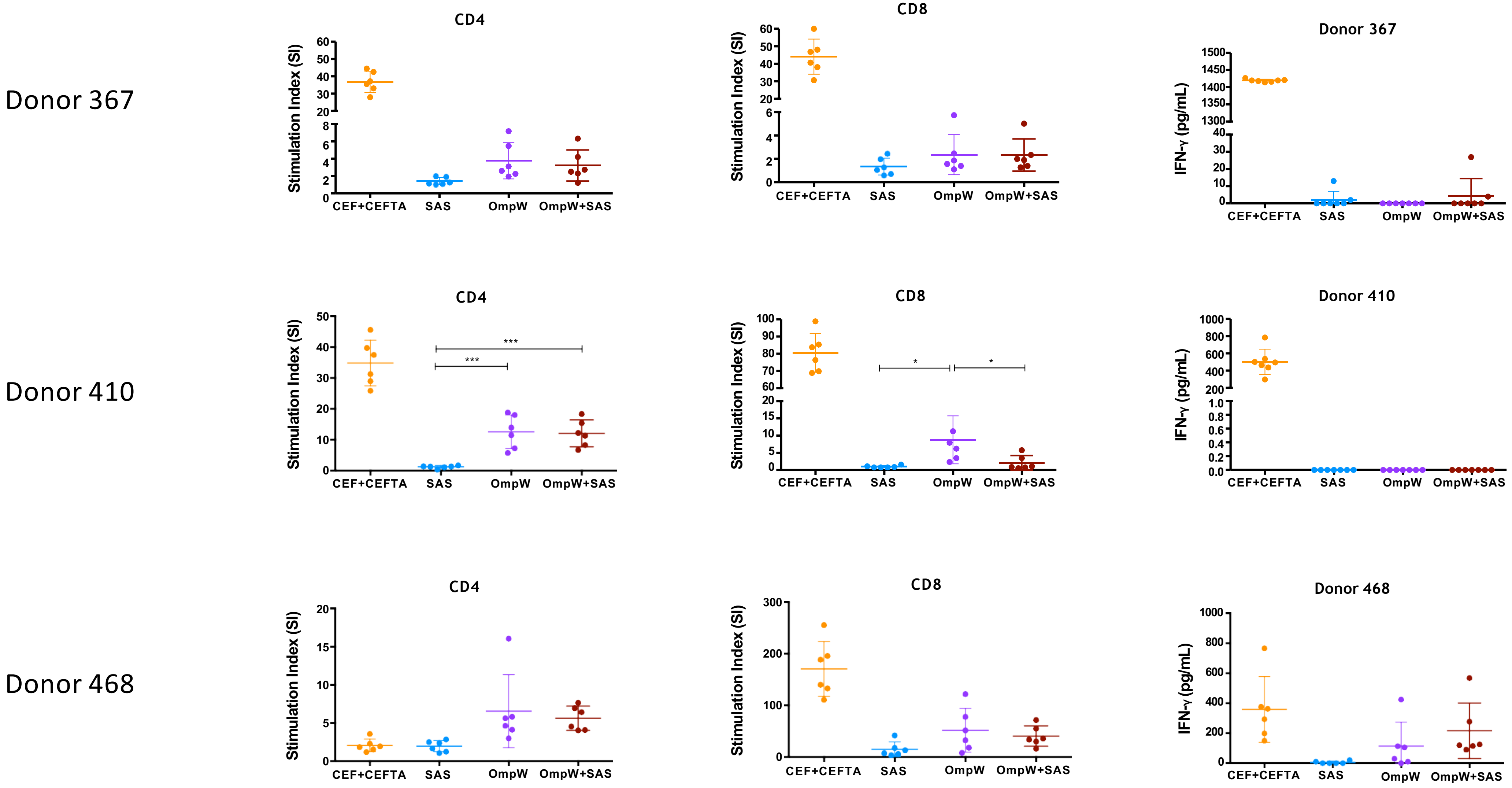

Donor 367
Donor 410
Donor 468
CD4
CD8
Donor 367
Donor 410
Donor 468
Stimulation Index (SI)
IFN-γ (pg/mL)
CEF+CEFTA
SAS
OmpW
OmpW+SAS
***
*

Figure S18

Donor 758

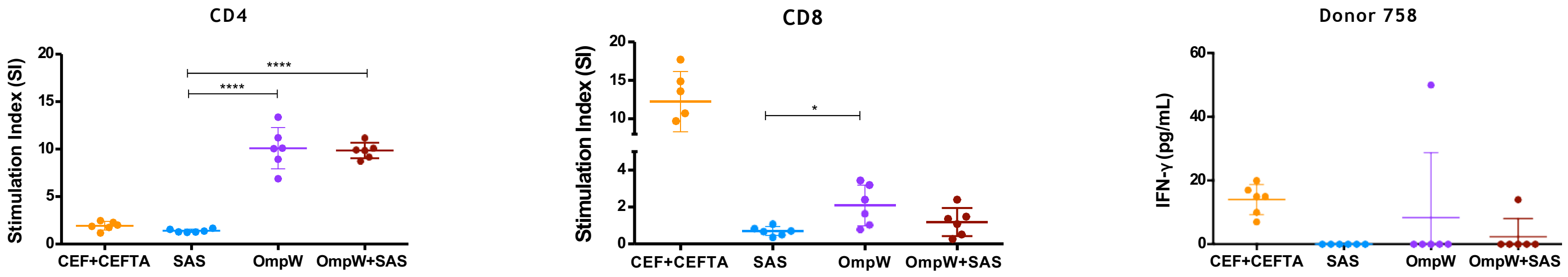

Donor 771

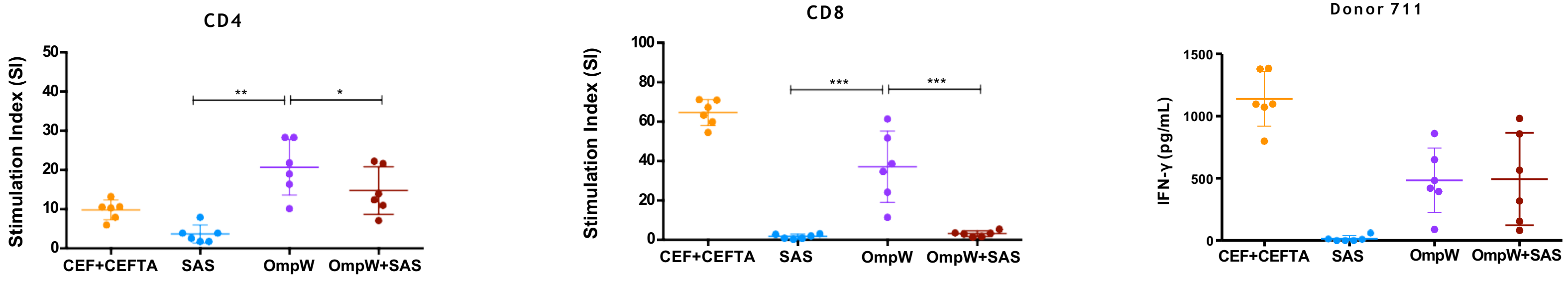

Figure S19

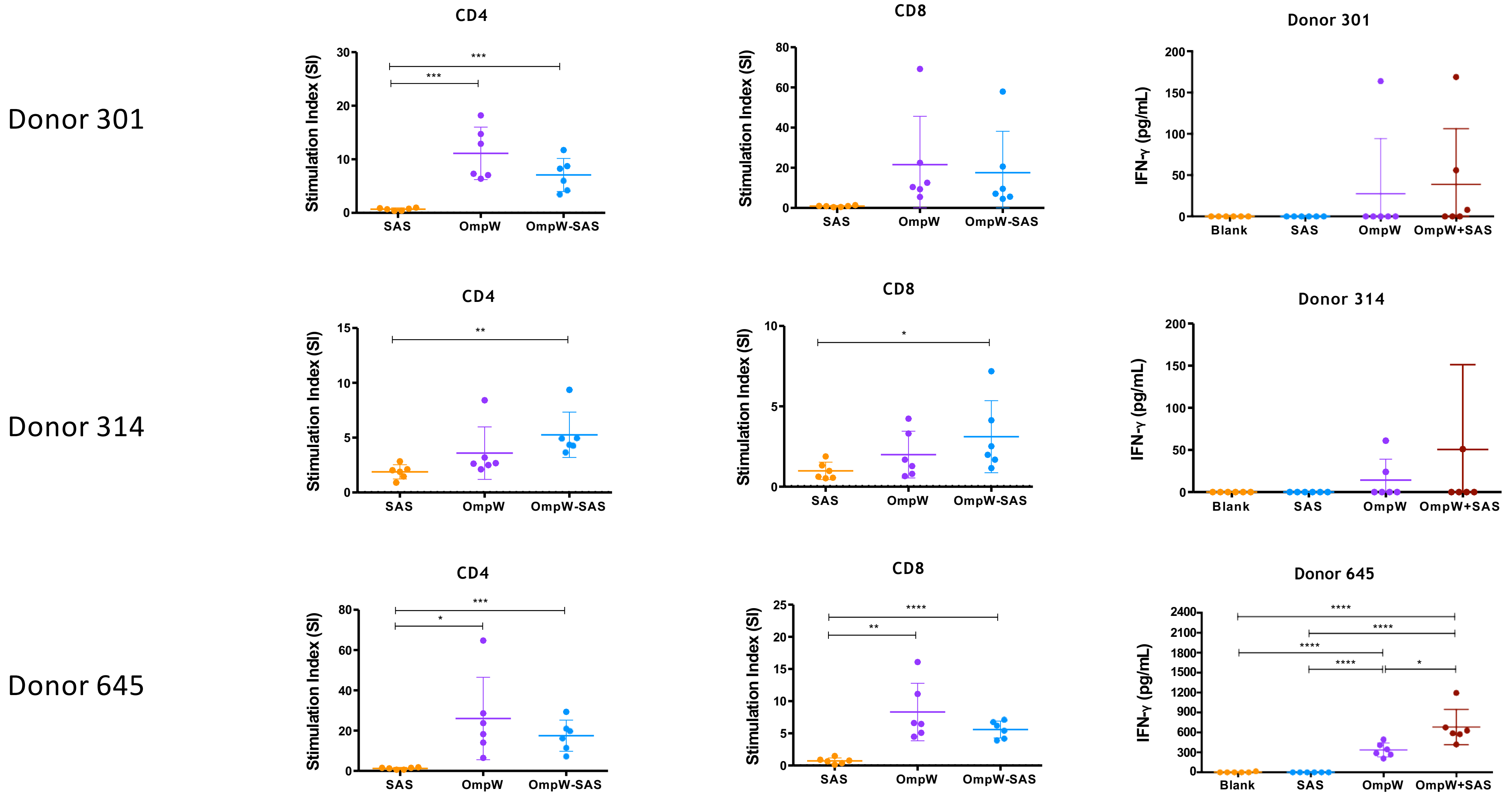
Donor 301
Donor 314
Donor 645
CD4
CD8
Donor 301
Donor 314
Donor 645
Stimulation Index (SI)
IFN-γ (pg/mL)
SAS
OmpW
OmpW-SAS
Blank
OmpW+SAS

Figs. S12-S19 Proliferation of $CD4^+$ and $CD8^+$ T cell populations in 23 healthy donor PBMCs by BpOmpW or SAS-adjuvanted BpOmpW, measured as stimulation index. IFN-γ production (pg/mL) in the harvested supernatant of 23 proliferation assays. SAS: human PBMCs exposed to adjuvant alone (Orange circles, Control). OmpW: human PBMCs exposed to BpOmpW alone. OmpW-SAS: human PBMCs exposed to BpOmpW + SAS adjuvant. Asterisks denote significant differences according to a two-tailed t-test. The levels of significance are represented as follows: (*, $p < 0.05$); (**, $p < 0.01$,); (***, $p < 0.001$); (****, $p < 0.0001$) .
